# Supplementary material for: The importance of species interactions in eco-evolutionary community dynamics under climate change
Source: Nat Commun. 2021 Aug 6;12:4759. doi: 10.1038/s41467-021-24977-x (PMC8346578; doi:10.1038/s41467-021-24977-x)
Supplement: Supplementary file 1 — Supplementary Information [file 41467_2021_24977_MOESM1_ESM.pdf]

The importance of species interactions in eco-evolutionary community dynamics under climate change
Supplementary Information

Anna Åkesson, Alva Curtsdotter, Anna Eklöf, Bo Ebenman,
Jon Norberg, György Barabás

1 Quantitative genetic recursion in space

Let S species be distributed across L habitat patches, with possible migration in between. Each species i is characterized by its density N_i^k and trait distribution $p_i^k(z)$ in patch k. Here z measures a unidimensional quantitative trait of interest; $N_i^k p_i^k(z)\mathrm{d}z$ is then the density of species i's individuals in patch k whose phenotype values fall between z and $z + \mathrm{d}z$. By definition,

$$\int p_i^k(z)\,\mathrm{d}z = 1 \tag{1}$$

at any moment of time. The mean trait of species i in patch k is

$$\mu_i^k = \int z p_i^k(z)\,\mathrm{d}z. \tag{2}$$

Our starting point is the framework of quantitative genetic recursion[1–5] in the weak selection limit[6]. We extend this approach by adding migration. The basic equation giving the change in the density distribution of species i in patch k reads

$$\tilde{N}_i^k \tilde{p}_i^k(z) = W_i^k(z) N_i^k p_i^k(z) + \sum_{l=1}^{L} M_i^{kl} N_i^l p_i^l(z) - \sum_{l=1}^{L} M_i^{lk} N_i^k p_i^k(z), \tag{3}$$

where the tilde denotes values after selection and migration, but before reproduction. The first term on the right hand side describes selection via the fitness function $W_i^k(z)$. The second term is immigration from all other patches (therefore M_i^{kl} is the dispersal rate from patch l to patch k, with $M_i^{kk} = 0$ for all i and k). The final term is the loss of species i's trait z in patch k due to emigration from the focal patch.

For the underlying genetics, we assume a large number of loci each contributing a very small additive effect to the trait[7,8]. In this infinitesimal model, all trait distributions $p_i^k(z)$ are normal with a variance σ_i^2 that does not change in response to selection:

$$p_i^k(z) = \frac{1}{\sigma_i\sqrt{2\pi}} \exp\left(-\frac{(z-\mu_i^k)^2}{2\sigma_i^2}\right). \tag{4}$$

The change in mean trait from one generation to the next is given by the breeder's equation[9]:

$$\hat{\mu}_i^k - \mu_i^k = h_i^2(\tilde{\mu}_i^k - \mu_i^k), \tag{5}$$

where the hat denotes values in the next generation, and $\tilde{\mu}_i^k$ denotes the trait mean after selection and migration, but before reproduction. After reproduction, $\hat{N}_i^k = \tilde{N}_i^k$, and $\hat{p}_i^k$ is given by Eq. 4 with $\hat{\mu}_i^k$ calculated from the breeder's equation[5].

In the weak selection limit, the fitness $W_i^k(z)$ of species i's phenotype z in patch k can be written

$$W_i^k(z) = 1 + s r_i^k(z), \tag{6}$$

where s is a small parameter, and $r_i^k(z)$ is the per capita growth rate of species i's phenotype z in patch k, defined by ecological interactions. The migration rates are similarly written as

$$M_i^{kl} = s m_i^{kl}. \tag{7}$$

To obtain the dynamics of the densities, we integrate Eq. 3 across z:

$$\tilde{N}_i^k \int \tilde{p}_i^k \,\mathrm{d}z = N_i^k \int W_i^k(z) p_i^k(z)\,\mathrm{d}z + \sum_{l=1}^{L} M_i^{kl} N_i^l \int p_i^l(z)\,\mathrm{d}z - \sum_{l=1}^{L} M_i^{lk} N_i^k \int p_i^k(z)\,\mathrm{d}z. \tag{8}$$

Using Eq. 1 and $\tilde{N}_i^k = \hat{N}_i^k$:

$$\hat{N}_i^k = N_i^k \int W_i^k(z) p_i^k(z)\,\mathrm{d}z + \sum_{l=1}^{L} M_i^{kl} N_i^l - \sum_{l=1}^{L} M_i^{lk} N_i^k. \tag{9}$$

In the weak selection limit (Eqs. 6 and 7),

$$\hat{N}_i^k = N_i^k \int \Big[1 + s r_i^k(z)\Big] p_i^k(z)\,\mathrm{d}z + s\sum_{l=1}^{L} m_i^{kl} N_i^l - s\sum_{l=1}^{L} m_i^{lk} N_i^k, \tag{10}$$

which, using Eq. 1, is written as

$$\hat{N}_i^k = N_i^k + s N_i^k \int r_i^k(z) p_i^k(z)\,\mathrm{d}z + s\sum_{l=1}^{L} m_i^{kl} N_i^l - s\sum_{l=1}^{L} m_i^{lk} N_i^k. \tag{11}$$

Subtracting N_i^k and dividing by s leads to

$$\frac{\hat{N}_i^k - N_i^k}{s} = N_i^k \int r_i^k(z) p_i^k(z)\,\mathrm{d}z + \sum_{l=1}^{L} m_i^{kl} N_i^l - \sum_{l=1}^{L} m_i^{lk} N_i^k. \tag{12}$$

With appropriate scaling[5], this can be written in differential equation form when s is sufficiently small:

$$\frac{\mathrm{d}N_i^k}{\mathrm{d}t} = N_i^k \int r_i^k(z) p_i^k(z)\,\mathrm{d}z + \sum_{l=1}^{L} m_i^{kl} N_i^l - \sum_{l=1}^{L} m_i^{lk} N_i^k. \tag{13}$$

To obtain the equation for the change in trait means, we write $\tilde{\mu}_i^k$ by rearranging Eq. 3:

$$\tilde{p}_i^k = \frac{W_i^k(z) N_i^k p_i^k(z) + \sum_{l=1}^{L} M_i^{kl} N_i^l p_i^l(z) - \sum_{l=1}^{L} M_i^{lk} N_i^k p_i^k(z)}{\hat{N}_i^k}. \tag{14}$$

We use Eq. 9 to expand the denominator:

$$\tilde{p}_i^k = \frac{W_i^k(z) N_i^k p_i^k(z) + \sum_{l=1}^{L} M_i^{kl} N_i^l p_i^l(z) - \sum_{l=1}^{L} M_i^{lk} N_i^k p_i^k(z)}{N_i^k \int W_i^k(z) p_i^k(z)\,\mathrm{d}z + \sum_{l=1}^{L} M_i^{kl} N_i^l - \sum_{l=1}^{L} M_i^{lk} N_i^k}. \tag{15}$$

We multiply both sides by z and integrate. Using Eq. 2, we get

$$\tilde{\mu}_i^k = \frac{N_i^k \int z W_i^k(z) p_i^k(z)\,\mathrm{d}z + \sum_{l=1}^{L} M_i^{kl} N_i^l \mu_i^l - \sum_{l=1}^{L} M_i^{lk} N_i^k \mu_i^k}{N_i^k \int W_i^k(z) p_i^k(z)\,\mathrm{d}z + \sum_{l=1}^{L} M_i^{kl} N_i^l - \sum_{l=1}^{L} M_i^{lk} N_i^k}. \tag{16}$$

In the weak selection limit (Eqs. 6 and 7), this reads

$$\tilde{\mu}_i^k = \frac{N_i^k \int z(1 + s r_i^k(z)) p_i^k(z)\,\mathrm{d}z + s\sum_{l=1}^{L} m_i^{kl} N_i^l \mu_i^l - s\sum_{l=1}^{L} m_i^{lk} N_i^k \mu_i^k}{N_i^k \int (1 + s r_i^k(z)) p_i^k(z)\,\mathrm{d}z + s\sum_{l=1}^{L} m_i^{kl} N_i^l - s\sum_{l=1}^{L} m_i^{lk} N_i^k}. \tag{17}$$

Using Eqs. 1 and 2, we get

$$\tilde{\mu}_i^k = \frac{N_i^k \mu_i^k + s N_i^k \int z r_i^k(z) p_i^k(z)\,\mathrm{d}z + s\sum_{l=1}^{L} m_i^{kl} N_i^l \mu_i^l - s\sum_{l=1}^{L} m_i^{lk} N_i^k \mu_i^k}{N_i^k + s N_i^k \int r_i^k(z) p_i^k(z)\,\mathrm{d}z + s\sum_{l=1}^{L} m_i^{kl} N_i^l - s\sum_{l=1}^{L} m_i^{lk} N_i^k}. \tag{18}$$

Simplifying by N_i^k and factoring out s from both the numerator and denominator:

$$\tilde{\mu}_i^k = \frac{\mu_i^k + s\left[\int z r_i^k(z) p_i^k(z)\,\mathrm{d}z + \sum_{l=1}^{L} m_i^{kl} \frac{N_i^l}{N_i^k} \mu_i^l - \sum_{l=1}^{L} m_i^{lk} \mu_i^k\right]}{1 + s\left[\int r_i^k(z) p_i^k(z)\,\mathrm{d}z + \sum_{l=1}^{L} m_i^{kl} \frac{N_i^l}{N_i^k} - \sum_{l=1}^{L} m_i^{lk}\right]}. \tag{19}$$

Taylor expanding around $s = 0$ to first order, we get

$$\begin{aligned}\tilde{\mu}_i^k = \mu_i^k + s\Bigg[&\int z r_i^k(z) p_i^k(z)\,\mathrm{d}z + \sum_{l=1}^{L} m_i^{kl} \frac{N_i^l}{N_i^k} \mu_i^l - \sum_{l=1}^{L} m_i^{lk} \mu_i^k \\ &-\mu_i^k\left(\int r_i^k(z) p_i^k(z)\,\mathrm{d}z + \sum_{l=1}^{L} m_i^{kl} \frac{N_i^l}{N_i^k} - \sum_{l=1}^{L} m_i^{lk}\right)\Bigg] + \mathcal{O}(s^2),\end{aligned} \tag{20}$$

where $\mathcal{O}(s^2)$ means terms of order s^2 or higher. Simplification yields

$$\tilde{\mu}_i^k = \mu_i^k + s\left[\int (z - \mu_i^k) r_i^k(z) p_i^k(z)\,\mathrm{d}z + \sum_{l=1}^{L} m_i^{kl} \frac{N_i^l}{N_i^k} (\mu_i^l - \mu_i^k)\right] + \mathcal{O}(s^2). \tag{21}$$

Substituting this expression into the breeder's equation (Eq. 5):

$$\hat{\mu}_i^k - \mu_i^k = h_i^2 s\left[\int (z - \mu_i^k) r_i^k(z) p_i^k(z)\,\mathrm{d}z + \sum_{l=1}^{L} m_i^{kl} \frac{N_i^l}{N_i^k} (\mu_i^l - \mu_i^k)\right]. \tag{22}$$

After dividing both sides by s and performing the $s \to 0$ limit with appropriate scaling[5], we get the differential equation approximation

$$\frac{\mathrm{d}\mu_i^k}{\mathrm{d}t} = h_i^2\left[\int (z - \mu_i^k) r_i^k(z) p_i^k(z)\,\mathrm{d}z + \sum_{l=1}^{L} m_i^{kl} \frac{N_i^l}{N_i^k} (\mu_i^l - \mu_i^k)\right]. \tag{23}$$

2 Per capita growth rates and species-level dynamics

We model a trophic community in space, with L distinct habitat patches. Each patch experiences an average temperature, which is increasing through time due to climate change. The evolvable trait z is identified with temperature tolerance: an individual with trait z achieves maximal intrinsic growth in a habitat with temperature z. Local population dynamics, excluding migration, are governed by four processes: temperature-dependent intrinsic growth, intra- and interspecific competition, growth due to consumption, and loss due to being consumed. The per capita growth rate of of species i's phenotype z in patch k is written

$$r_i^k(z) = r_{0,i}^k(z) - \sum_{j=1}^{S} N_j^k \int a_{ij}^k(z,z') p_j^k(z')\,\mathrm{d}z' + \sum_{j=1}^{S} \epsilon_i F_{ij}^k - \sum_{j=1}^{S} N_j^k F_{ji}^k / N_i^k, \tag{24}$$

where, in patch k, $r_{0,i}^k(z)$ is the intrinsic growth rate of species i's phenotype z, $a_{ij}^k(z,z')$ are competition coefficients between species i's phenotype z and species j's phenotype z', ϵ_i is species i's resource conversion efficiency, and F_{ij}^k is the feeding rate of species i on j. It has the form

$$F_{ij}^k = \frac{q_i W_{ij} \omega_{ij} N_j^k}{1 + q_i H_i \sum_{s=1}^{S} W_{is} \omega_{is} N_s^k}, \tag{25}$$

where q_i is species i's attack rate, W_{ij} is the adjacency matrix of the feeding network ($W_{ij} = 1$ if i eats j and 0 otherwise), ω_{ij} is the relative consumption rate of i on j (we assume an equal split across resources, so that $\omega_{ij} = [\text{number of resources}]^{-1}$ for all consumers), and H_i is species i's handling time.

Substituting the growth rate in Eq. 24 into Eqs. 13 and 23 yields

$$\begin{aligned}\frac{\mathrm{d}N_i^k}{\mathrm{d}t} &= N_i^k \int r_{0,i}^k(z) p_i^k(z)\,\mathrm{d}z - N_i^k \int \sum_{j=1}^{S} N_j^k \left[\int a_{ij}^k(z,z') p_j^k(z')\,\mathrm{d}z'\right] p_i^k(z)\,\mathrm{d}z \\ &\quad + N_i^k \int \left[\sum_{j=1}^{S} \epsilon_i F_{ij}^k - \sum_{j=1}^{S} N_j^k F_{ji}^k / N_i^k\right] p_i^k(z)\,\mathrm{d}z + \sum_{l=1}^{L} m_i^{kl} N_i^l - \sum_{l=1}^{L} m_i^{lk} N_i^k\end{aligned} \tag{26}$$

for the population densities, and

$$\begin{aligned}\frac{\mathrm{d}\mu_i^k}{\mathrm{d}t} &= h_i^2 \int (z-\mu_i^k) r_{0,i}^k(z) p_i^k(z)\,\mathrm{d}z - h_i^2 \int (z-\mu_i^k) \sum_{j=1}^{S} N_j^k \left[\int a_{ij}^k(z,z') p_j^k(z')\,\mathrm{d}z'\right] p_i^k(z)\,\mathrm{d}z \\ &\quad + h_i^2 \int \left[\sum_{j=1}^{S} \epsilon_i F_{ij}^k - \sum_{j=1}^{S} N_j^k F_{ji}^k / N_i^k\right] (z-\mu_i^k) p_i^k(z)\,\mathrm{d}z + h_i^2 \sum_{l=1}^{L} m_i^{kl} \frac{N_i^l}{N_i^k} (\mu_i^l - \mu_i^k)\end{aligned} \tag{27}$$

for the trait means. Rearranging, we get

$$\begin{aligned}\frac{\mathrm{d}N_i^k}{\mathrm{d}t} &= N_i^k \int r_{0,i}^k(z) p_i^k(z)\,\mathrm{d}z - N_i^k \sum_{j=1}^{S} N_j^k \iint p_i^k(z) a_{ij}^k(z,z') p_j^k(z')\,\mathrm{d}z'\mathrm{d}z \\ &\quad + N_i^k \left[\sum_{j=1}^{S} \epsilon_i F_{ij}^k - \sum_{j=1}^{S} N_j^k F_{ji}^k / N_i^k\right] \int p_i^k(z)\,\mathrm{d}z + \sum_{l=1}^{L} m_i^{kl} N_i^l - \sum_{l=1}^{L} m_i^{lk} N_i^k,\end{aligned} \tag{28}$$

$$\begin{aligned}\frac{\mathrm{d}\mu_i^k}{\mathrm{d}t} &= h_i^2 \int (z-\mu_i^k) r_{0,i}^k(z) p_i^k(z)\,\mathrm{d}z - h_i^2 \sum_{j=1}^{S} N_j^k \iint (z-\mu_i^k) p_i^k(z) a_{ij}^k(z,z') p_j^k(z')\,\mathrm{d}z'\mathrm{d}z \\ &\quad + h_i^2 \left[\sum_{j=1}^{S} \epsilon_i F_{ij}^k - \sum_{j=1}^{S} N_j^k F_{ji}^k / N_i^k\right] \int (z-\mu_i^k) p_i^k(z)\,\mathrm{d}z + h_i^2 \sum_{l=1}^{L} m_i^{kl} \frac{N_i^l}{N_i^k} (\mu_i^l - \mu_i^k).\end{aligned} \tag{29}$$

By Eq. 1, the integral in the third term of Eq. 28 is simply 1. In turn, the integral in the third term of Eq. 29 is zero, because the integrand is a product of an odd and an even function in $z - \mu_i^k$. This leads to

$$\frac{\mathrm{d}N_i^k}{\mathrm{d}t} = N_i^k \int r_{0,i}^k(z)p_i^k(z)\,\mathrm{d}z - N_i^k \sum_{j=1}^{S} N_j^k \iint p_i^k(z)a_{ij}^k(z,z')p_j^k(z')\,\mathrm{d}z'\mathrm{d}z \tag{30}$$
$$+ \sum_{j=1}^{S} \epsilon_i N_i^k F_{ij}^k - \sum_{j=1}^{S} N_j^k F_{ji}^k + \sum_{l=1}^{L} m_i^{kl} N_i^l - \sum_{l=1}^{L} m_i^{lk} N_i^k,$$

$$\frac{\mathrm{d}\mu_i^k}{\mathrm{d}t} = h_i^2 \int (z-\mu_i^k) r_{0,i}^k(z)p_i^k(z)\,\mathrm{d}z - h_i^2 \sum_{j=1}^{S} N_j^k \iint (z-\mu_i^k)p_i^k(z)a_{ij}^k(z,z')p_j^k(z')\,\mathrm{d}z'\mathrm{d}z \tag{31}$$
$$+ h_i^2 \sum_{l=1}^{L} m_i^{kl} \frac{N_i^l}{N_i^k}(\mu_i^l - \mu_i^k).$$

Introducing the definitions

$$b_i^k = \int r_{0,i}^k(z)p_i^k(z)\,\mathrm{d}z, \tag{32}$$

$$\alpha_{ij}^k = \iint p_i^k(z)a_{ij}^k(z,z')p_j^k(z')\,\mathrm{d}z'\mathrm{d}z, \tag{33}$$

$$g_i^k = \int (z-\mu_i^k) r_{0,i}^k(z)p_i^k(z)\,\mathrm{d}z, \tag{34}$$

$$\beta_{ij}^k = \iint (z-\mu_i^k)p_i^k(z)a_{ij}^k(z,z')p_j^k(z')\,\mathrm{d}z'\mathrm{d}z, \tag{35}$$

Eqs. 30-31 read

$$\frac{\mathrm{d}N_i^k}{\mathrm{d}t} = N_i^k b_i^k - N_i^k \sum_{j=1}^{S} \alpha_{ij}^k N_j^k + \sum_{j=1}^{S} \epsilon_i N_i^k F_{ij}^k - \sum_{j=1}^{S} N_j^k F_{ji}^k + \sum_{l=1}^{L} m_i^{kl} N_i^l - N_i^k \sum_{l=1}^{L} m_i^{lk}, \tag{36}$$

$$\frac{\mathrm{d}\mu_i^k}{\mathrm{d}t} = h_i^2 g_i^k - h_i^2 \sum_{j=1}^{S} \beta_{ij}^k N_j^k + h_i^2 \sum_{l=1}^{L} m_i^{kl} \frac{N_i^l}{N_i^k}(\mu_i^l - \mu_i^k), \tag{37}$$

with F_{ij}^k given by Eq. 25.

3 Model parameterization

Here we present the detailed derivation and parameterization of each model component. Numerical values of the model's parameters, along with their units and short descriptions, are collected in Supplementary Tables 1-2.

3.1 Temperature-dependent intrinsic growth rates

Following Amarasekare & Johnson[10], we model the temperature-dependence of $r_{0,i}^k(z)$ using the Gaussian form

$$r_{0,i}^k(z) = A_i^k \exp\left(-\frac{(T^k - z)^2}{2(w_i^k)^2}\right) - \kappa_i, \tag{38}$$

where κ_i is a mortality rate for species i, $A_i^k - \kappa_i$ is the maximum intrinsic growth that can be achieved for species i in patch k, T^k is the temperature in patch k, and w_i^k is the species- and patch-specific width of the growth curve.

The w_i^k are not constant, but co-evolve with species' mean temperature optima μ_i^k. Within reasonable limits (such that the tolerance widths remain positive), this can be modeled using the following linear relationship:

$$w_i^k = b_w - a_w \mu_i^k, \tag{39}$$

where a_w and b_w are positive constant parameters. This creates a trend of increasingly broader curves for lower temperature optima, corresponding to the observed correlation between tolerance width and latitude[11,12]. For determining A_i^k, we use the observation that there is a tradeoff between the width and maximum height of temperature optima. This phenomenon is brought about by higher annual temperature variability at higher latitudes: since reaction norms are nonlinear functions of temperature, the optimal response to the mean temperature is not the same as the mean optimal response, due to Jensen's inequality[10]. This also means that the distance between experienced and optimal temperatures is larger at higher latitudes, where climate tends to be more variable[13,14]. However, our model does not take annual temperature fluctuations into account, which means that the observed optimum shift cannot evolve as in the work of Amarasekare & Johnson[10]. Instead, we imposed this tradeoff by assigning a smaller A_i^k to wider temperature adaptations at low μ_i^k values (Eq. 39), mimicking the fact that populations at higher latitudes are adapted to more variable environments and thus also living at temperatures further away from those that would yield maximum growth in a stable environment. The tradeoff is implemented as

$$A_i^k = \frac{\varrho_i}{w_i^k} = \frac{\varrho_i}{b_w - a_w \mu_i^k}, \tag{40}$$

where ϱ_i is a parameter modulating the shape of the tradeoff. Substituting the parameter specifications of Eqs. 39-40 into Eq. 38 gives

$$r_{0,i}^k(z) = \left(\frac{\varrho_i}{b_w - a_w \mu_i^k} \right) \exp\left(-\frac{(T^k - z)^2}{2(b_w - a_w \mu_i^k)^2} \right) - \kappa_i. \tag{41}$$

This form of the tradeoff, and its particular parameterization (Supplementary Table 1), were chosen so the resulting growth curves would qualitatively mimick those found empirically[13,14]. Supplementary Figure 1 shows what the growth rates of Eq. 41 look like, in six species equally spaced along a temperature gradient, both for consumers and resources.

To obtain the species-level parameters b_i^k and g_i^k from this growth rate, we substitute Eqs. 4 and 41 into Eqs. 32 and 34:

$$b_i^k = \int \left[\left(\frac{\varrho_i}{b_w - a_w \mu_i^k} \right) \exp\left(-\frac{(T^k - z)^2}{2(b_w - a_w \mu_i^k)^2} \right) - \kappa_i \right] \frac{\exp\left(-\frac{(z-\mu_i^k)^2}{2\sigma_i^2} \right)}{\sigma_i \sqrt{2\pi}} \, \mathrm{d}z, \tag{42}$$

$$g_i^k = \int (z - \mu_i^k) \left[\left(\frac{\varrho_i}{b_w - a_w \mu_i^k} \right) \exp\left(-\frac{(T^k - z)^2}{2(b_w - a_w \mu_i^k)^2} \right) - \kappa_i \right] \frac{\exp\left(-\frac{(z-\mu_i^k)^2}{2\sigma_i^2} \right)}{\sigma_i \sqrt{2\pi}} \, \mathrm{d}z. \tag{43}$$

The integrals can be evaluated directly, leading to the final forms

$$b_i^k = \left(\frac{\varrho_i}{b_w - a_w \mu_i^k} \right) \frac{b_w - a_w \mu_i^k}{\sqrt{(b_w - a_w \mu_i^k)^2 + \sigma_i^2}} \exp\left(-\frac{(T^k - \mu_i^k)^2}{2[(b_w - a_w \mu_i^k)^2 + \sigma_i^2]} \right) - \kappa_i, \tag{44}$$

$$g_i^k = \left(\frac{\varrho_i}{b_w - a_w \mu_i^k} \right) \frac{\sigma_i^2 (b_w - a_w \mu_i^k)(T^k - \mu_i^k)}{[(b_w - a_w \mu_i^k)^2 + \sigma_i^2]^{3/2}} \exp\left(-\frac{(T^k - \mu_i^k)^2}{2[(b_w - a_w \mu_i^k)^2 + \sigma_i^2]} \right). \tag{45}$$

3.2 Competition coefficients

We only model competition between resource species. Consumers also compete but only indirectly, via shared resources. This means that the competition kernel $a_{ij}^k(z, z')$ is zero unless both i and

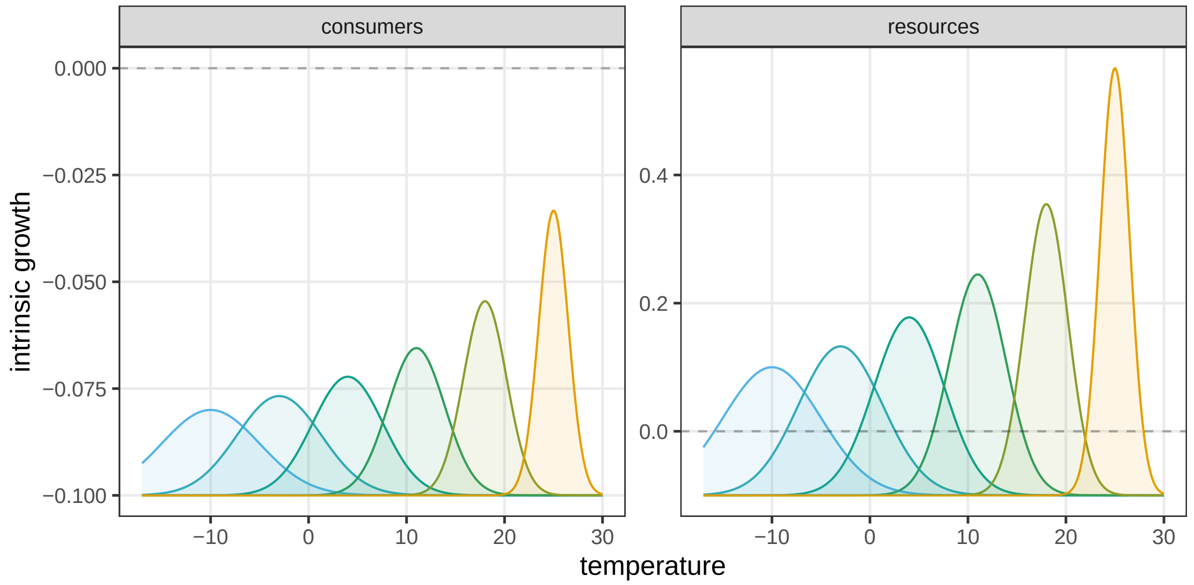

Supplementary Figure 1: Tradeoff between the height and breadth of temperature optima, for phenotypes of six consumer (left) and six resource species (right). Colors indicate species, with cooler shades standing for more cold-adapted ones. The dashed line indicates zero growth; therefore the $r_{0,i}^k$ of consumer species is always a mortality rate, which must be compensated by consumption. For resources, $r_{0,i}^k$ is positive unless the local temperature is too far from the optimum for that species. In this figure, $\kappa_i = 0.1$ for all species, $\varrho_i = 0.1$ for all consumers, and $\varrho_i = 0.1$ for all resources. In our simulations, to make sure that the model's predicted community patterns are not an artifact of the above precise tradeoff shapes, the ϱ_i were randomized in a $\pm 10\%$ range of these values (Supplementary Table 1).

j refer to resource species. When they do, we use two different kernel forms. One only depends on species identity, and is independent of trait or patch value:

$$a_{ij}^k(z, z') = a_{ij}. \tag{46}$$

Applying Eqs. 33 and 35 to obtain α_{ij} and β_{ij}, we get

$$\alpha_{ij}^k = a_{ij} \iint p_i^k(z) p_j^k(z') \, \mathrm{d}z' \mathrm{d}z = a_{ij}, \tag{47}$$

$$\beta_{ij}^k = a_{ij} \iint (z - \mu_i^k) p_i^k(z) p_j^k(z') \, \mathrm{d}z' \mathrm{d}z = 0. \tag{48}$$

In the first equation, the integrals evaluate to 1 because of Eq. 4, while in the second equation, integration with respect to z involves the product of an odd and an even function in $z - \mu_i^k$ and is therefore zero. The constant a_{ij} values are sampled randomly (Supplementary Table 1), in a way that ensures intraspecific competition being always stronger than interspecific competition.

The second form of the competition kernel we use depends on the difference between phenotypic values:

$$a_{ij}^k(z, z') = \exp\left(-\frac{(z - z')^2}{\eta^2} \right), \tag{49}$$

where η is the competition width, determining how distant two phenotypes must be for competition to be significantly reduced between them. Here α_{ij}^k and β_{ij}^k can be calculated by direct

integration using Eqs. 33 and 35:

$$\alpha_{ij}^k = \frac{\eta}{\sqrt{2\sigma_i^2 + 2\sigma_j^2 + \eta^2}} \exp\left(-\frac{(\mu_i^k - \mu_j^k)^2}{2\sigma_i^2 + 2\sigma_j^2 + \eta^2} \right), \tag{50}$$

$$\beta_{ij}^k = -\frac{2\eta\sigma_i^2(\mu_i^k - \mu_j^k)}{(2\sigma_i^2 + 2\sigma_j^2 + \eta^2)^{3/2}} \exp\left(-\frac{(\mu_i^k - \mu_j^k)^2}{2\sigma_i^2 + 2\sigma_j^2 + \eta^2} \right). \tag{51}$$

This form of the competition kernel facilitates trait divergence even within a single patch, since it may be profitable for a species to specialize on a suboptimal temperature to thus weaken interspecific competition and coexist locally. Biologically, this mechanism is explained by having microhabitats within each patch, with higher and lower local temperatures than the patch average. A species with a higher or lower temperature optimum than the average can exploit these microhabitats better than the locally dominant species, and therefore persist in the patch as a whole—though it will not be able to achieve as much growth due to the relatively limited availability of such microhabitats.

3.3 Feeding network

We model two strict trophic levels, with S resource and S consumer species. The bipartite network of feeding connections W_{ij} (with $W_{ij} = 1$ if i eats j and 0 otherwise) is generated as follows. First, both resources and consumers are labeled consecutively, based on their initial temperature adaptations: resource 1 / consumer 1 are the most cold-adapted, and resource S / consumer S the most warm-adapted. Next, we always put a feeding link between consumer i and resource i. Finally, each consumer is randomly linked to four other resource species. This yields a feeding network where every consumer is connected to five resources altogether.

3.4 Genetic and environmental variances

Genetic variances $V_{G,i}$ are randomly drawn for each species, while the environmental variances $V_{E,i}$ are fixed (Supplementary Table 2). We assume that there is no epistatic or dominance variance, which means that the total phenotypic variance, σ_i^2, is the sum of the additive genetic and environmental variances: $\sigma_i^2 = V_{G,i} + V_{E,i}$. The heritability of species i's trait value is, by definition, the ratio of genetic to total phenotypic variance[9], so we have

$$h_i^2 = \frac{V_{G,i}}{V_{G,i} + V_{E,i}} = \frac{V_{G,i}}{\sigma_i^2}. \tag{52}$$

Our model has no demographic stochasticity. This means we are assuming that even "low" population densities are sufficiently high to neglect genetic and ecological drift. This, however, can introduce problems with artifactual evolutionary rescue, where an unrealistically tiny population evolves to the point where it can finally grow, thus surviving and establishing itself even though it was bound to go extinct. To control for this artifact, we introduced a reduction in species' genetic variances whenever their local population densities dropped below the threshold N_c. Below this threshold, genetic variances were multiplied by a factor $Q(N_i^k/N_c)$, where

$$Q(x) = \begin{cases} 0 & \text{if } x < 0, \\ 10x^3 - 15x^4 + 6x^5 & \text{if } 0 \le x \le 1, \\ 1 & \text{if } x > 1. \end{cases} \tag{53}$$

This is a smoothed step function: it is 0 for $x < 0$, increases monotonically to one at $x = 1$, and stays equal to 1 for $x > 1$ (Supplementary Figure 2). It is constructed to be twice continuously differentiable for all x.

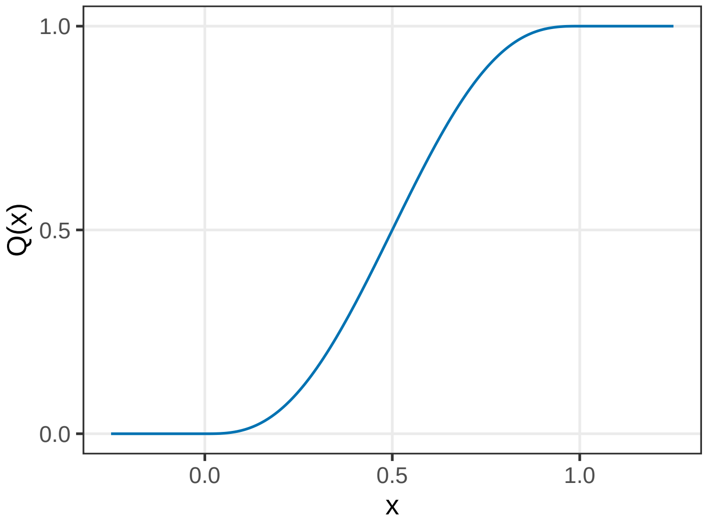

Supplementary Figure 2: The smooth step function $Q(x)$, defined by Eq. 53. It is constructed to be piecewise polynomial and twice continuously differentiable everywhere.

This means that heritabilities were also modified below N_c, being instead given by the patch-dependent expression

$$h_i^2 = \frac{Q(N_i^k/N_c)V_{G,i}}{Q(N_i^k/N_c)V_{G,i} + V_{E,i}}. \tag{54}$$

3.5 Spatial structure and dispersal rates

We discretize latitudinal position into L equidistant patches: patch 1 is at the pole, patch L at the equator, with the rest linearly spaced in between. Migration happens between adjacent patches with a species-specific rate d_i:

$$m_i^{kl} = \begin{cases} d_i & \text{if } k = l \pm 1, \\ 0 & \text{otherwise.} \end{cases} \qquad (k, l = 1, \ldots, L) \tag{55}$$

To the approximation that the two hemispheres of Earth are symmetric with respect to their temperature profiles, the pole-to-equator spatial profile could be repeated from equator to the other pole again (in reverse), and then wrapped around the whole planet. Due to this symmetry and periodicity, the range going from pole to equator already contains all information, and is the only part that needs to be modeled, provided one implements appropriate boundary conditions to account for the periodicity. Specifically, for patches 1 and L, the following replacements are made compared to Eqs. 36-37:

$$\frac{\mathrm{d}N_i^1}{\mathrm{d}t} \to \frac{\mathrm{d}N_i^1}{\mathrm{d}t} + m_i^{1,2} N_i^2 - m_i^{2,1} N_i^1, \tag{56}$$

$$\frac{\mathrm{d}N_i^L}{\mathrm{d}t} \to \frac{\mathrm{d}N_i^L}{\mathrm{d}t} + m_i^{L,L-1} N_i^{L-1} - m_i^{L-1,L} N_i^L, \tag{57}$$

$$\frac{\mathrm{d}\mu_i^1}{\mathrm{d}t} \to \frac{\mathrm{d}\mu_i^1}{\mathrm{d}t} + h_i^2 m_i^{1,2} \frac{N_i^2}{N_i^1} (\mu_i^2 - \mu_i^1), \tag{58}$$

$$\frac{\mathrm{d}\mu_i^L}{\mathrm{d}t} \to \frac{\mathrm{d}\mu_i^L}{\mathrm{d}t} + h_i^2 m_i^{L,L-1} \frac{N_i^{L-1}}{N_i^L} (\mu_i^{L-1} - \mu_i^L). \tag{59}$$

3.6 Climate model

Let x denote latitudinal position, measured as the latitudinal distance from the north pole. The temperature at position x and time t is given by $T(x,t)$. The local temperature T^k in patch k is the one at the latitude corresponding to patch k. The components of the climate change model are as follows:

1. Temperature is initially constant in each patch, for a burn-in period from $t_0 = -4000$ to 0 years. The initial average temperature $T_0(x)$ is $T_{\min}$ at the pole, $T_{\max}$ at the equator (Supplementary Table 1), and linearly extrapolated in between:
$$T_0(x) = (T_{\max} - T_{\min})\frac{x}{X} + T_{\min}, \tag{60}$$
where X is the distance from pole to equator, measured in the same units as x.

2. Climate change starts at $t = 0$, after the burn-in period from $t = t_0$ to $t = 0$. In each patch, the qualitative shape of the relative temperature rise is given by the smoothed step function $Q(t/t_E)$ (Eq. 53), where t_E is the end of the climate change period.

3. Climate change stops after $t_E = 300$ years.

4. The extent of the temperature increase depends on latitude. This latitudinal trend is predicted with high confidence for the northern hemisphere for the next century, and is observed in all of IPCC's emission scenarios[15]. We assume that the latitude-dependence of the final temperature increase (at t_E) is linear:
$$C_E(x) = (C_{\min} - C_{\max})\frac{x}{X} + C_{\max}, \tag{61}$$
where $C_{\max}$ is the maximum temperature increase (at the pole) and $C_{\min}$ is the minimum increase (at the equator). The values of $C_{\max}$ and $C_{\min}$ (Supplementary Table 1) are based on region-specific predictions of increase in temperature by 2100 CE, in combination with estimates giving approximate increase by 2300 CE, for the IPCC intermediate emission scenario[15].

Considering all the above, the time-dependence of the temperature profile $T(x,t)$ is written compactly as
$$T(x,t) = T_0(x) + C_E(x)Q(t/t_E). \tag{62}$$

3.7 Initial conditions

Both for resource species $1, 2, \dots, S$ and for consumer species $1, 2, \dots, S$, the initial temperature adaptations $\mu_i^k(t_0)$ are determined by the initial temperature profile such that species 1 is adapted to the polar temperatures, species S to equatorial temperatures, and all other species are linearly spaced in between:
$$\mu_i^k(t_0) = T_0(i/S) = (T_{\max} - T_{\min})\frac{i}{S} + T_{\min} \tag{63}$$
at all spatial locations where the species is initially present. Initial population densities are centered at the location to which the species is best adapted, and are normally distributed around this point in space:
$$N_i^k(t_0) = \exp\left(-\frac{(\mu_i^k(t_0) - T_0(k/L))^2}{2 \times 2^2}\right). \tag{64}$$

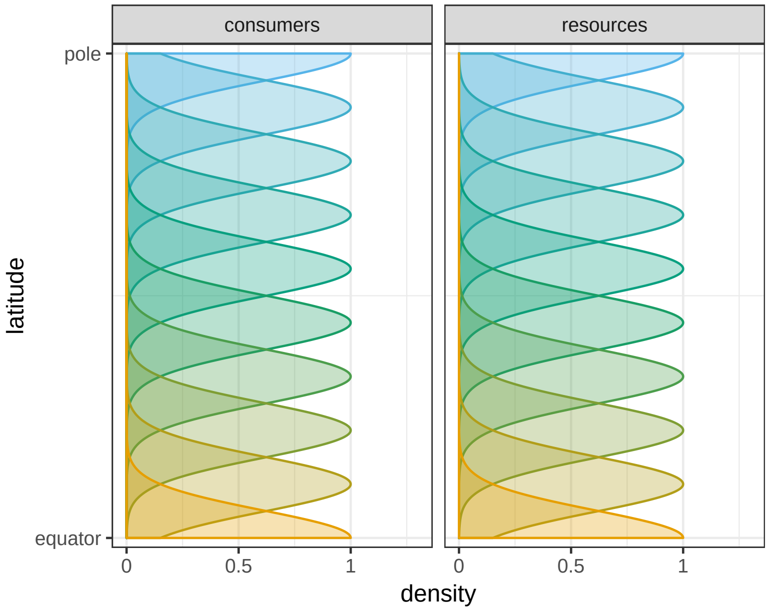

Supplementary Figure 3: Initial density distribution of ten consumer (left) and ten resource species (right) along the latitudinal gradient (ordinate). The abscissa shows the population density at the corresponding latitude. Colors denote species, with cooler shades standing for more cold-adapted species.

4 Community response capacity

Let there be S species distributed across L patches, evolving according to our model. We denote the relative density of species i in patch k at time t by $n_i^k(t)$:

$$n_i^k(t) = \frac{N_i^k(t)}{\sum_{j=1}^{S} N_j^k(t)}, \tag{65}$$

and the community-weighted mean trait in patch k at time t by $\bar{\mu}^k(t)$:

$$\bar{\mu}^k(t) = \sum_{i=1}^{S} n_i^k(t)\mu_i^k(t). \tag{66}$$

We then define the trait lag in patch k, $\mathcal{A}^k(t)$, as

$$\mathcal{A}^k(t) = T^k(t) - \bar{\mu}^k(t), \tag{67}$$

where $T^k(t)$ is the local temperature in patch k at time t. This quantity measures the extent to which traits in a local community match their environment, on average. $\mathcal{A}^k(t)$ may be further averaged across patches and time, yielding

$$\mathcal{A} = \frac{1}{L}\sum_{k=1}^{L}\frac{1}{t_E}\sum_{t=0}^{t_E}\mathcal{A}^k(t). \tag{68}$$

The time averaging moves from the onset to the end of climate change, i.e., between years 0 and $t_E = 300$. In turn, we define the density-weighted dispersion of mean trait values, $\mathcal{V}^k(t)$, as

$$\mathcal{V}^k(t) = \sum_{i=1}^{S} n_i^k(t)\left[\mu_i^k(t) - \bar{\mu}^k(t)\right]^2. \tag{69}$$

The more different species are in their trait means (with relatively more common species getting more weight for being different), the larger this value becomes. Again, we can average this quantity across time and patches to obtain

$$\mathcal{V} = \frac{1}{L} \sum_{k=1}^{L} \frac{1}{t_E} \sum_{t=0}^{t_E} \mathcal{V}^k(t). \tag{70}$$

$\mathcal{A}$ and $\mathcal{V}$ can be calculated for each community and plotted against one another, as was done in Figure 7 in the main text.

4.1 Trait dispersion and species richness

Regardless of the model analyzed, higher species richness results in higher trait dispersion overall (Supplementary Figure 4). However, this global trend is sometimes locally reversed for particular parameterizations—namely, when genetic variance is low and competition is temperature-dependent. This creates a version of Simpson's paradox [16], where the aggregated data show different trends than disaggregated pieces of it.

The extent of trait dispersion strongly depends on the mode of competition between resource species: if competition is temperature-dependent (Supplementary Figure 4, bottom two panels), trait dispersion is about an order of magnitude higher than otherwise (top two panels; note the difference in scale along the abscissas). This is in line with the fact that, with temperature-dependent competition, species can coexist by being sufficiently different from one another, by adapting to locally suboptimal temperatures which nevertheless reduces competition to tolerable levels. Without temperature-dependent competition, whether species adapt to have similar temperature optima has no consequence to how strongly they compete. Thus, if a set of species can coexist, they can do so at the local trait optimum. The presence of many species at the optimum naturally results in reduced trait dispersion (Eq. 69).

With temperature-dependent competition, high spatial dispersal ability results in higher trait dispersion (Supplementary Figure 4, bottom panels, blue and yellow). High genetic variance and low dispersal reduce both richness and dispersion in all models: the lack of dispersal means that each local community is more independent from its neighbors, at which point the locally readily adaptable species organize themselves into a stable community relatively quickly. This is known to lead to lower richness than equivalent communities without the ability to adapt—though these lower-richness communities are also more robust against environmental perturbations [5]. However, high genetic variance and dispersal ability versus low variance and dispersal shift position depending on whether competition is temperature-dependent.

5 Additional results

5.1 Community turnover

Species turnover in time is measured by the Jaccard distance. Let $\mathcal{S}(t)$ denote the set of species present at time t, and $|\mathcal{S}(t)|$ the number of elements in the set. The Jaccard distance $J(t_0, t)$ between the community state at some reference time t_0 and a later time t reads

$$J(t_0, t) = 1 - \frac{|\mathcal{S}(t_0) \cap \mathcal{S}(t)|}{|\mathcal{S}(t_0) \cup \mathcal{S}(t)|}, \tag{71}$$

where $\cap$ is the intersection and $\cup$ the union of two sets. This formula is agnostic about the spatial extent of a community: it can be applied to each patch separately, or the community as a whole. An in-between approach is to obtain regional turnover by applying Eq. 71 to the top third of all L patches (the polar region), the middle third (temperate region), and bottom third (tropical region). Supplementary Figure 5 shows how regional species compositions change in

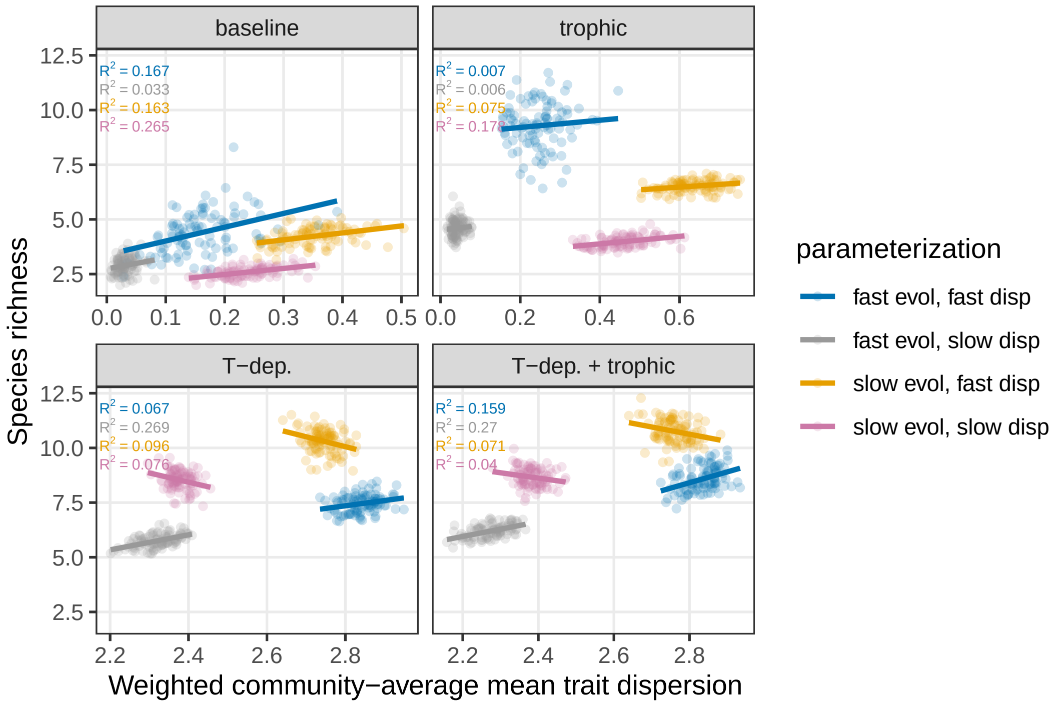

Supplementary Figure 4: Community average trait dispersion against species richness, averaged over the landscape and time from the beginning to the end of the climate change period, yielding a single number for every community (points). The sub-figures show communities in the four different models. Species' dispersal ability and available genetic variance (colors) are clustered in each sub-figure.

time, with the community state at the start of climate change playing the role of the reference community at t_0.

5.2 Beta diversity and rank-abundance curves

Beta diversity, the turnover in species diversity when moving from one local site to another within a larger regional community, is calculated following Whittaker's approach[17], where $\beta = \gamma/\alpha$. Here α accounts for local species diversity, and γ is the total species richness over all patches. The former, α, is calculated as in Figure 4 of the main text, with species mean per patch over the landscape. In turn, we obtain β as in the global patterns of Figure 6 of the main text. Supplementary Figure 6 shows how beta diversity changes over time for the resource species.

To display species' relative abundances, we obtained rank-abundance curves for resource species 7. The calculation is based on species' mean abundances over all patches and replicates, at the end of our simulations ($t = 2500$). Species are then ordered from highest to lowest abundance.

5.3 Generalist and specialist consumers

In our model, a consumer always feeds on the resource species with an initial corresponding temperature adaptation, and four additional randomly chosen species (Section 3.3). Here we relax this assumption and show result for generalist and specialist consumers. In the specialist case, each consumer feed on one single resource species (the one with corresponding initial temperature adaptation). In the generalist case, each consumer feeds on their matching resource, and is also randomly linked to $S/2 - 1$ other resources (rounded if necessary). For S even, this will lead to the feeding network having a connectance of 1/2.

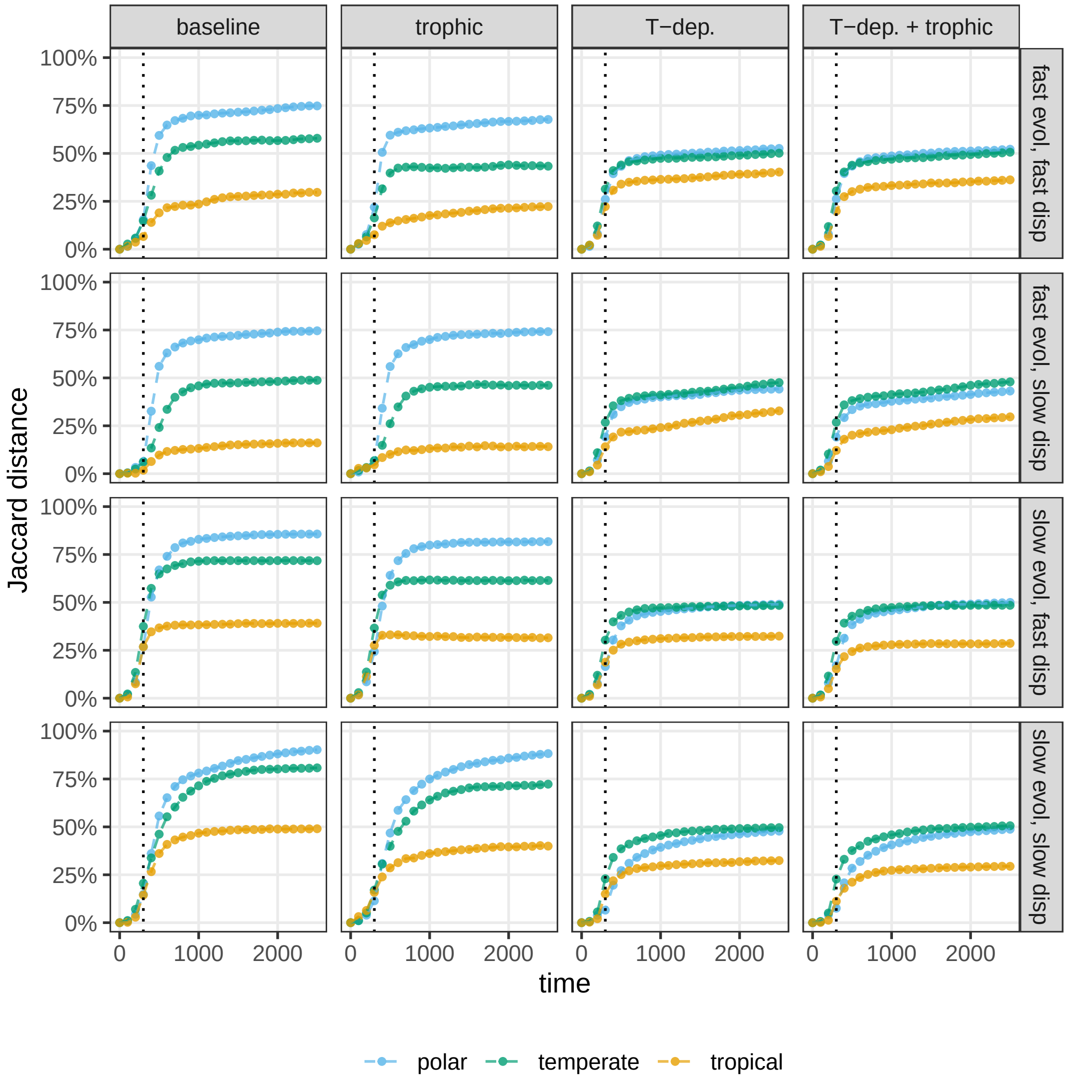

Supplementary Figure 5: Jaccard distance between the regional community composition of resource species at the start of climate change and subsequent times, in steps of 100 years (points; dashed lines connect them for better visibility). A value of 0% means that the same set of species is present as at the start, while 100% indicates a complete turnover in regional species composition. The Jaccard distance is averaged over patches (merged into regions, indicated by color) and replicates. Columns show different ecological models, rows various parameter combinations of average dispersal ability and available genetic variance.

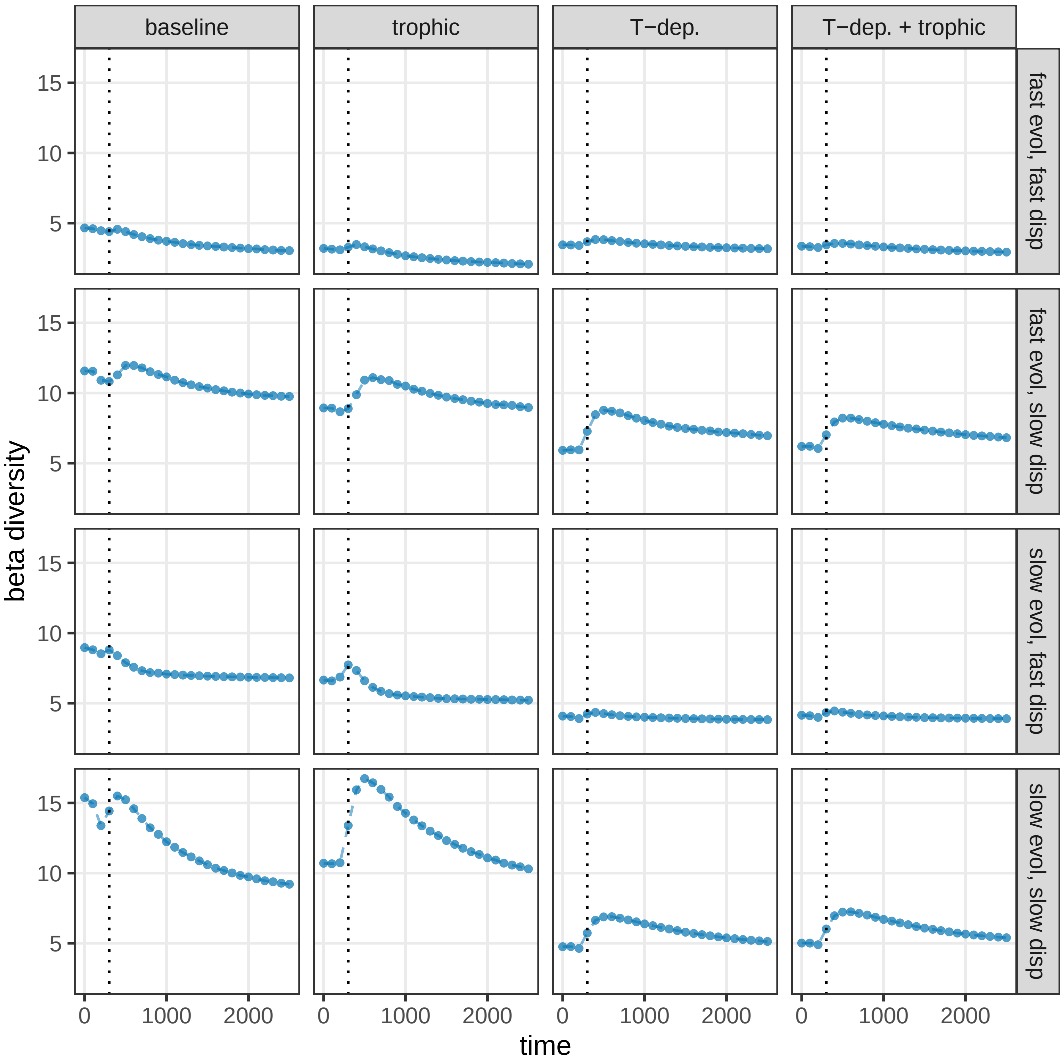

Supplementary Figure 6: Beta diversity as measured by the ratio of regional to local species diversity, at the start of climate change and subsequent times in steps of 100 years (points; dashed lines connect them for better visibility). A high beta diversity indicates that the difference between local and global species diversity is large. A low beta diversity indicates that species are evenly distributed across the landscape, with no great difference in local and global species diversity. The beta diversity is averaged over replicates. Columns show different ecological models, rows various parameter combinations of average dispersal ability and available genetic variance.

The patterns of our original setup are broadly retained with generalist and specialist consumers (Supplementary Figures 8-13). The difference is that greater resource specificity leads to increased diversity, both on the global and local scales. This is in line with the intuition that specialist predators help maintain resource diversity, since now each resource species is additionally regulated by an independent factor. However, even when predators are generalists, they have a positive effect on local and global resource diversity patterns—albeit this positive effect is diminished in comparison with the specialist case.

5.4 The influence of trophic interactions and temperature-dependent competition on local diversity

Figure 4 in the main text shows that while the effects of either trophic interactions or temperature-dependent competition on local diversity are quite strong, their combined effect is not very

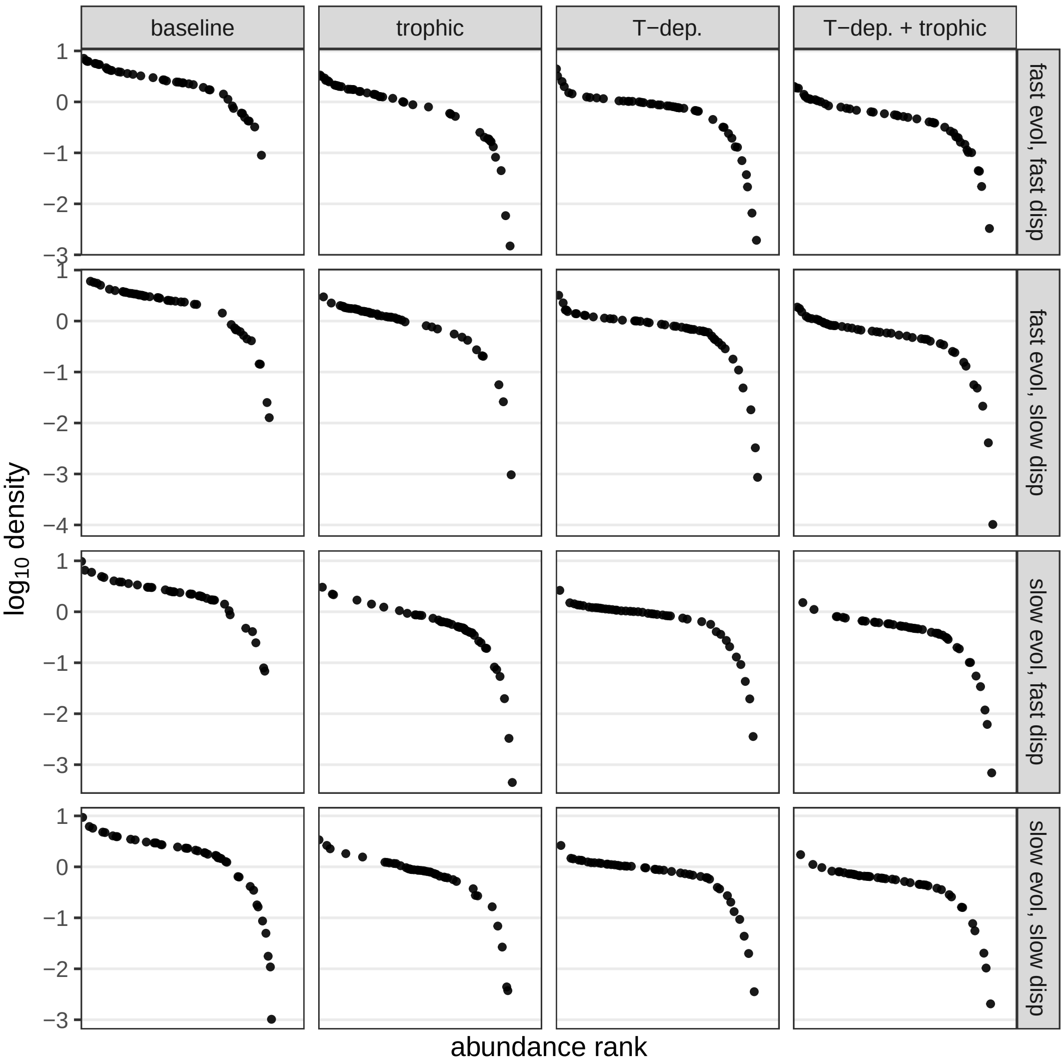

Supplementary Figure 7: Rank-abundance curves displaying relative species abundance. The relative abundance on the y-axis is shown on a $\log_{10}$ scale. The steeper the gradient of each curve, the greater difference in abundances between high-ranked and low-ranked species. A shallow gradient indicates that the abundances of species are similar. The calculation is based on species mean abundances over all patches and replicates, at the end of simulations ($t = 2500$). Columns show different ecological models, rows various parameter combinations of average dispersal ability and available genetic variance.

different from the effect of just one or the other mechanism in isolation. We can further elucidate this pattern with a 2-way ANOVA, and obtaining confidence intervals for the effect sizes of trophic interactions alone, temperature-dependent competition alone, and their interaction, using Tukey's test. We do so separately for each combination of parameter settings (high and low genetic variance and dispersal ability) in Supplementary Figures 14-17 below.

5.5 Zero dispersal

We have also repeated our analyses setting all dispersal rates to zero. In this case, we find narrower range breadths and lower local species diversity than in the presence of dispersal (Supplementary Figures 18-21). In terms of global species richness, global losses are mitigated by temperature-dependent competition also without dispersal. Trophic interactions do so as well, but only when available genetic variance is limited (slow evolution). By contrast, local diversity is promoted by trophic interactions alone but mostly for high levels of available genetic

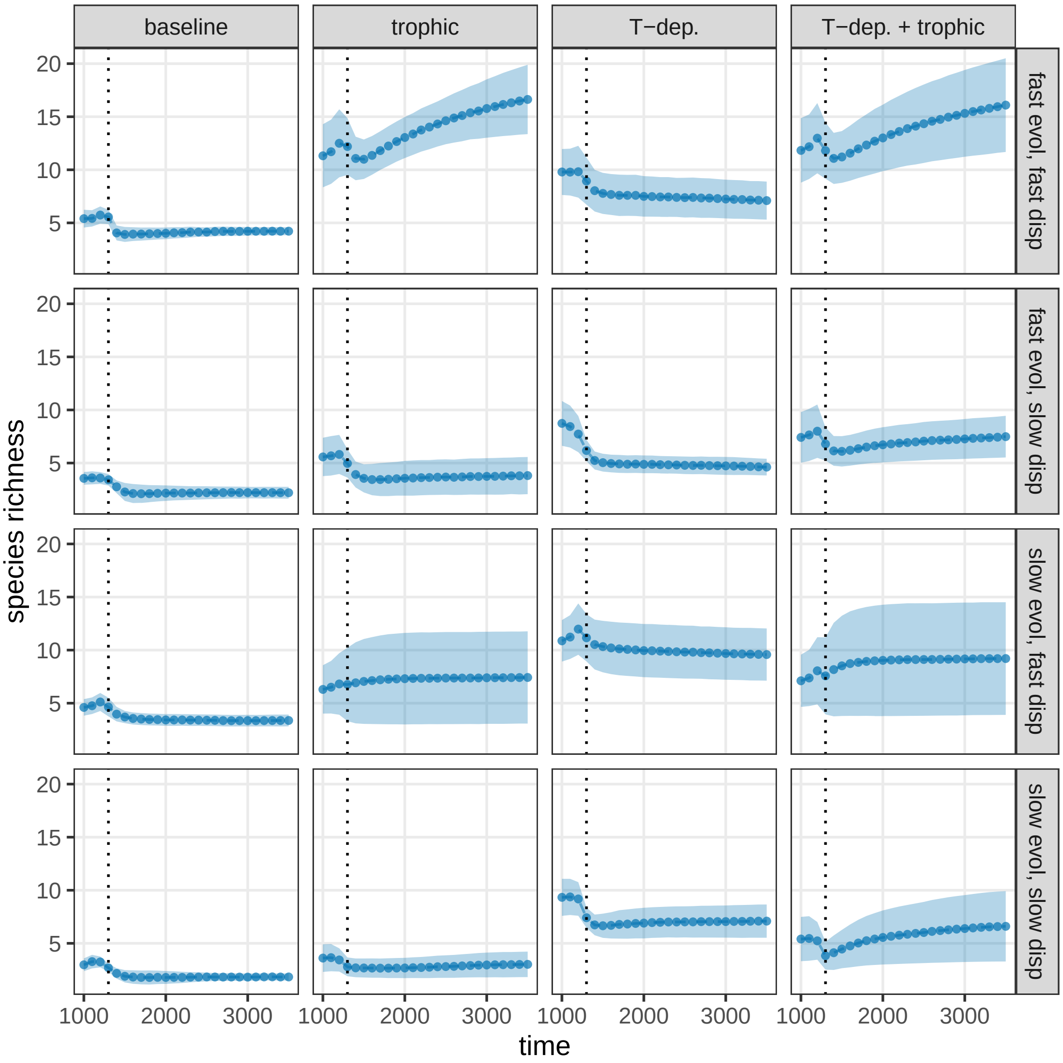

Supplementary Figure 8: As Figure 4 of the main text, but with specialist consumers.

variance. Both for local and global diversity, temperature-dependent competition has a stronger positive effect on diversity than trophic interactions alone.

Isolating patches by setting dispersal to zero allows us to contrast our results with some earlier ones, obtained for trophic dynamics under climate change but without dispersal. Similar to the findings of Osmond et al.[18], consumer presence in our model reduces resource densities (Figure 3). With consumer presence but without dispersal, we observe slightly boosted local resource richness and increased ranges, in comparison to what we see without consumers (Supplementary Figure 18-19). Contrasting with Osmond et al.[18], we observe a similar but marginal effect of consumer presence, with slightly fewer global losses when evolution is slow (Supplementary Figure 20).

Cortez & Yamamichi[19] focus on how evolution in prey, predators, or both, affect responses of predator populations to an increase in mortality. Both synergistic and antagonistic effects between prey and predator evolution appear, but compared to their scenario with no evolution at all, evolution of one or both species increases the predator mortality extinction threshold in most cases. What we observe is that higher levels of available genetic variance (leading to faster evolution) actually slightly decreases consumer persistence (Supplementary Figure 21). While this appears to contrast with the observed increase in mortality thresholds by Cortez & Yamamichi[19], they in fact have shown that consumer density can temporarily *in*crease as a

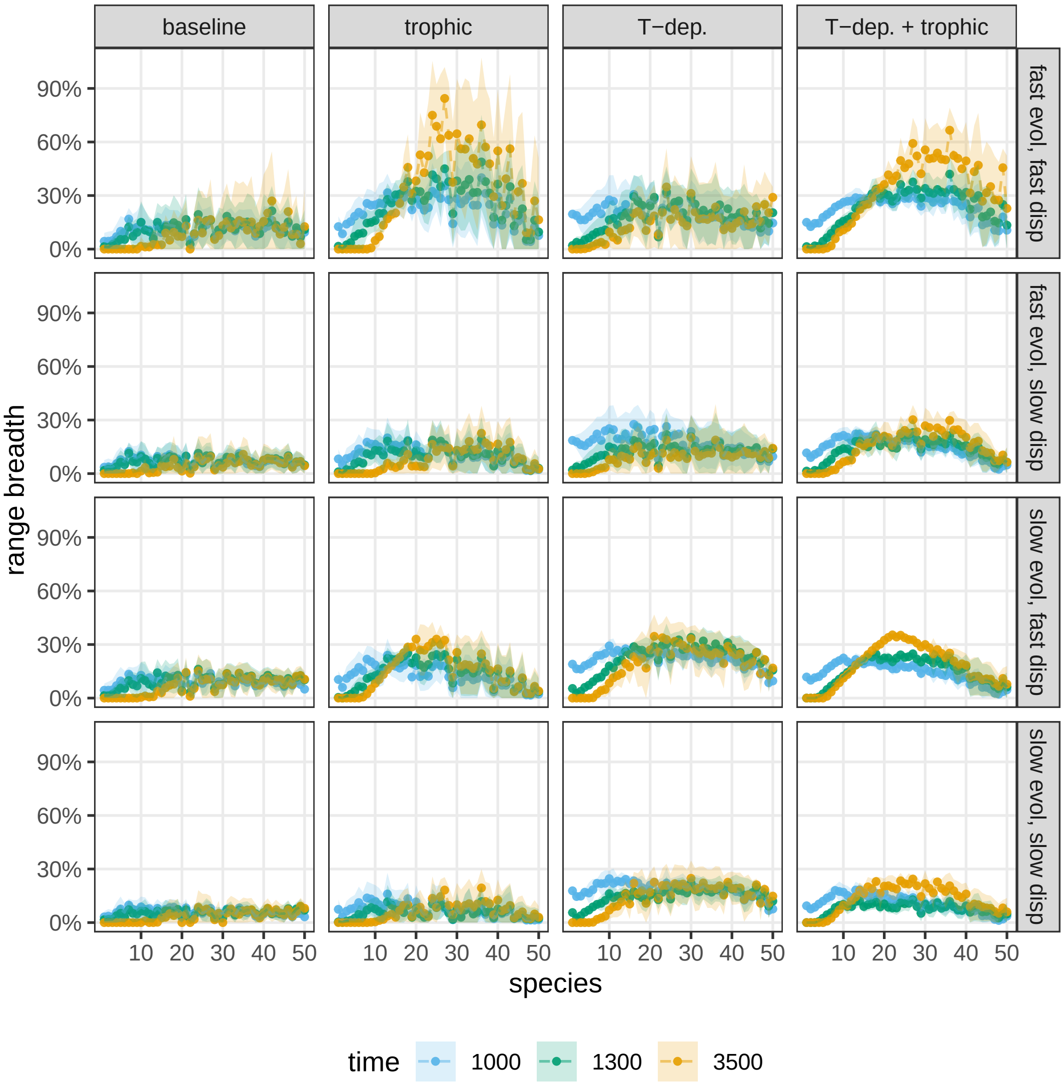

Supplementary Figure 9: As Figure 5 of the main text, but with specialist consumers.

function of increasing mortality (hydra effect). For this reason, the outcome of increasing the speed of evolution is not immediately obvious in our multispecies setting. The fact that we find a slight reduction in consumer persistence is therefore not necessarily inconsistent with their findings.

5.6 The effect of dispersal kernels

For the main results of this study, we used a simple, symmetric dispersal kernel in which species migrate with a given rate to the adjacent patches in both a southward and northward direction (Eq. 55). We also repeated our simulations using a Gaussian, symmetric dispersal kernel given by $\exp(-x^2/(2\sigma^2))$, where x is the distance between two patches and $\sigma = 0.02\sqrt{2/\pi}$ is chosen to keep the total rate of migration the same as with the original, nearest-neighbor dispersal. Our qualitative conclusions are robust to this change of dispersal mode (Supplementary Figures 22-24).

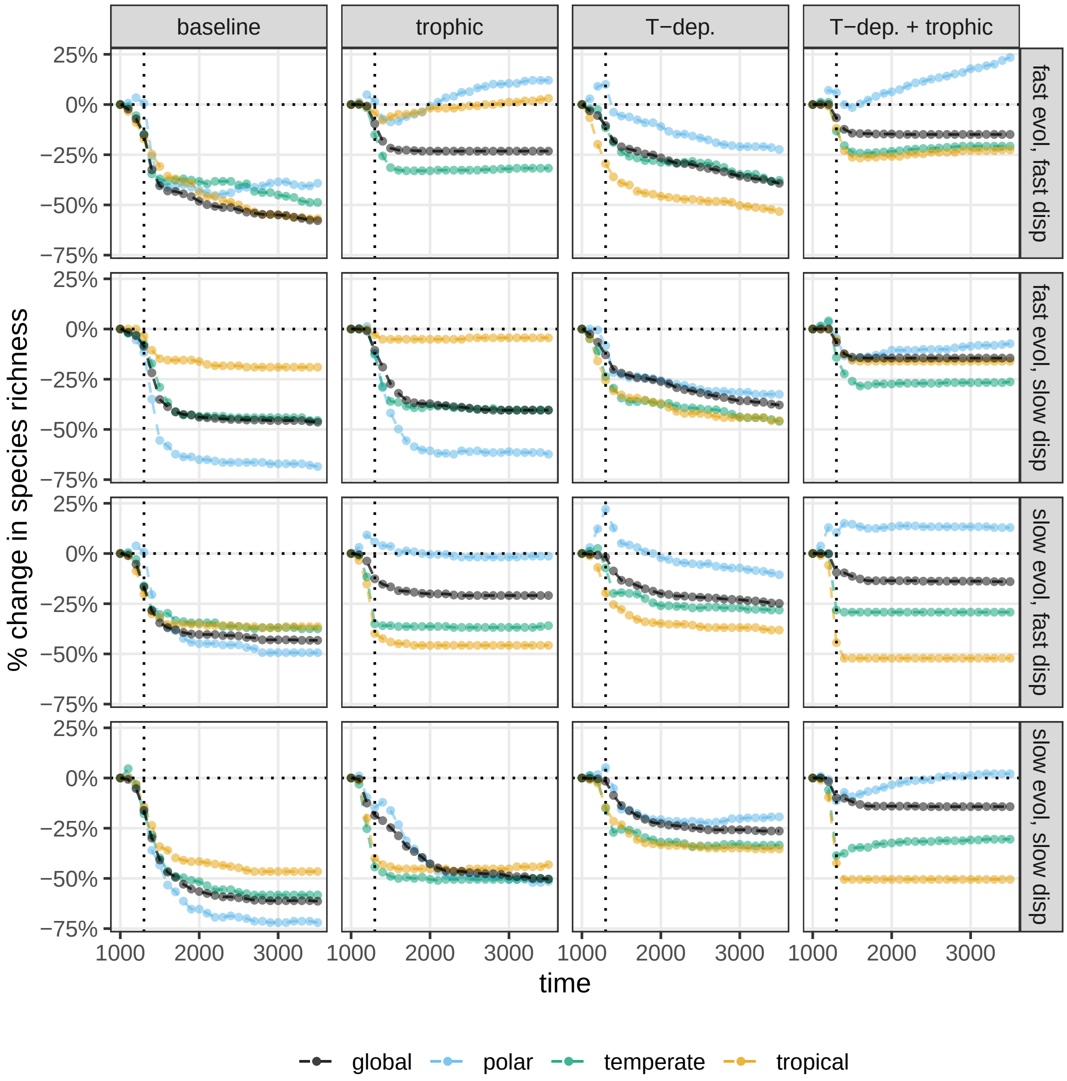

Supplementary Figure 10: As Figure 6 of the main text, but with specialist consumers.

We additionally explored asymmetric dispersal, by modifying Eq. 55 to introduce a northward or southward bias in movement:

$$m_i^{kl} = \begin{cases} d_i^+ & \text{if } k = l - 1, \\ d_i^- & \text{if } k = l + 1, \\ 0 & \text{otherwise.} \end{cases} \qquad (k, l = 1, \ldots, L) \tag{72}$$

Having $d_i^+ > d_i^-$ introduces a northward movement bias, while $d_i^+ < d_i^-$ introduces a southward bias. We chose $d_i^+ = 1.5d_i$, $d_i^- = 0.5d_i$ in the former and $d_i^+ = 0.5d_i$, $d_i^- = 1.5d_i$ in the latter case, where d_i is the original dispersal rate of the symmetric dispersal. Overall, asymmetry does not influence the community patterns much, independent of whether we have a northward bias (Supplementary Figures 25-27) or a southward bias (Supplementary Figures 28-30).

5.7 Competitive equivalence

We also performed a robustness analysis, contrasting our results with a model where species are competitively equivalent. This means that if species also had the same intrinsic growth rates, they would have equal fitness under all circumstances, and so neutral ecological drift

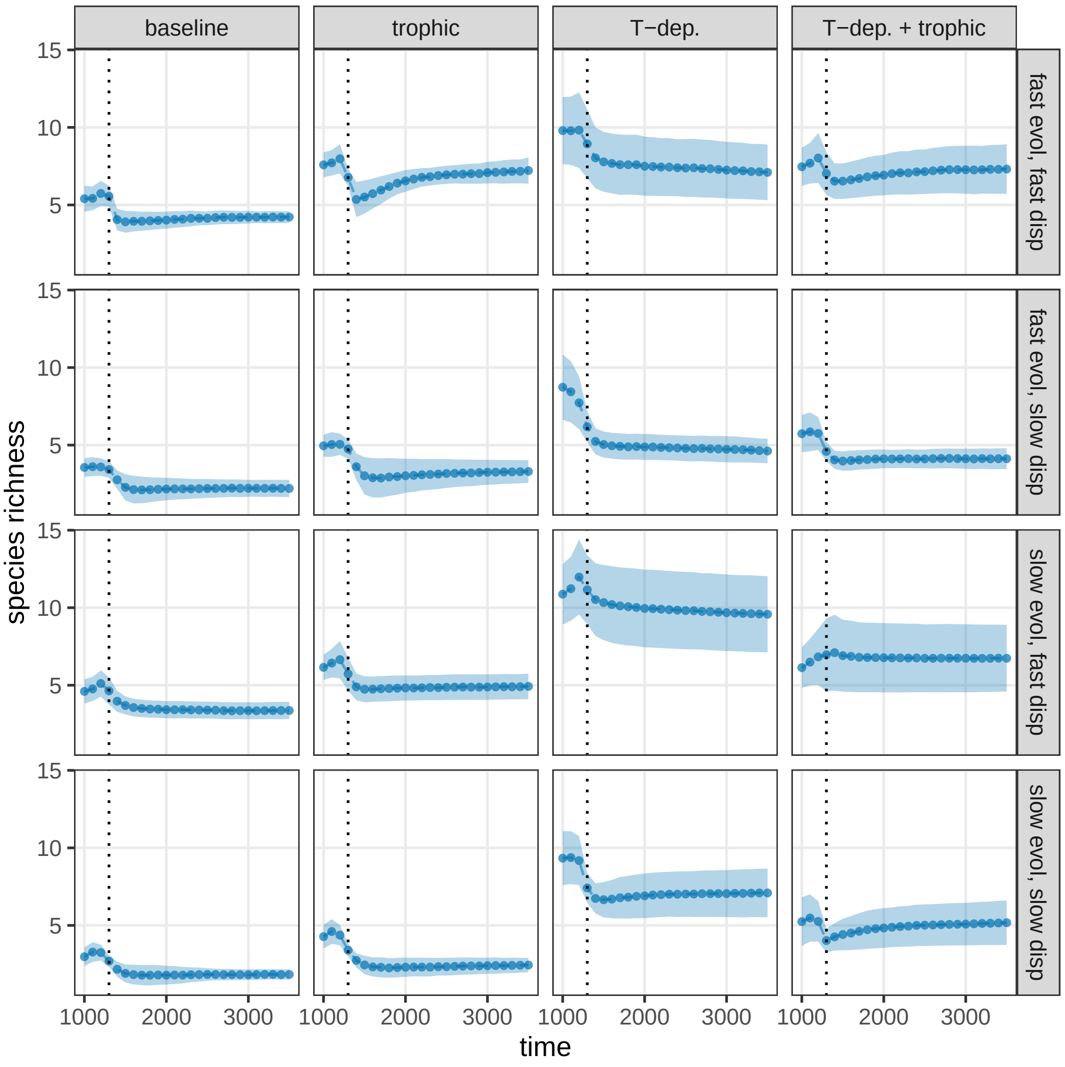

Supplementary Figure 11: As Figure 4 of the main text, but with generalist consumers.

would be the only process changing relative abundances. To create such a model, we have re-run our simulations with all intra- and interspecific interactions equal (species still retain the ability to adapt differently to temperature). We used a value of 0.2 for species' intra- and interspecific competition, since this value is the mean of the range of intraspecific competition coefficients used otherwise (Supplementary Table 1). As inclusion of both a second trophic level and temperature-dependent competition would break competitive equivalence, we only simulated our baseline model with these equal competition coefficients.

While the results are qualitatively similar to our original baseline model (Supplementary Figures 31-33), an important difference is that competitive equivalence leads to a 25-50% reduction in local species diversity. In comparison with our other models, with a second trophic level and/or temperature-dependent competition, local diversity is approximately 50-80% lower. However, global species richness is slightly higher in the temperate and tropical regions, in particular when evolution is slow. Overall, temperature-dependent competition mitigates global extinctions significantly (global losses of 20-25%), when compared with the baseline model with competitive equivalence (global losses of 40-50%).

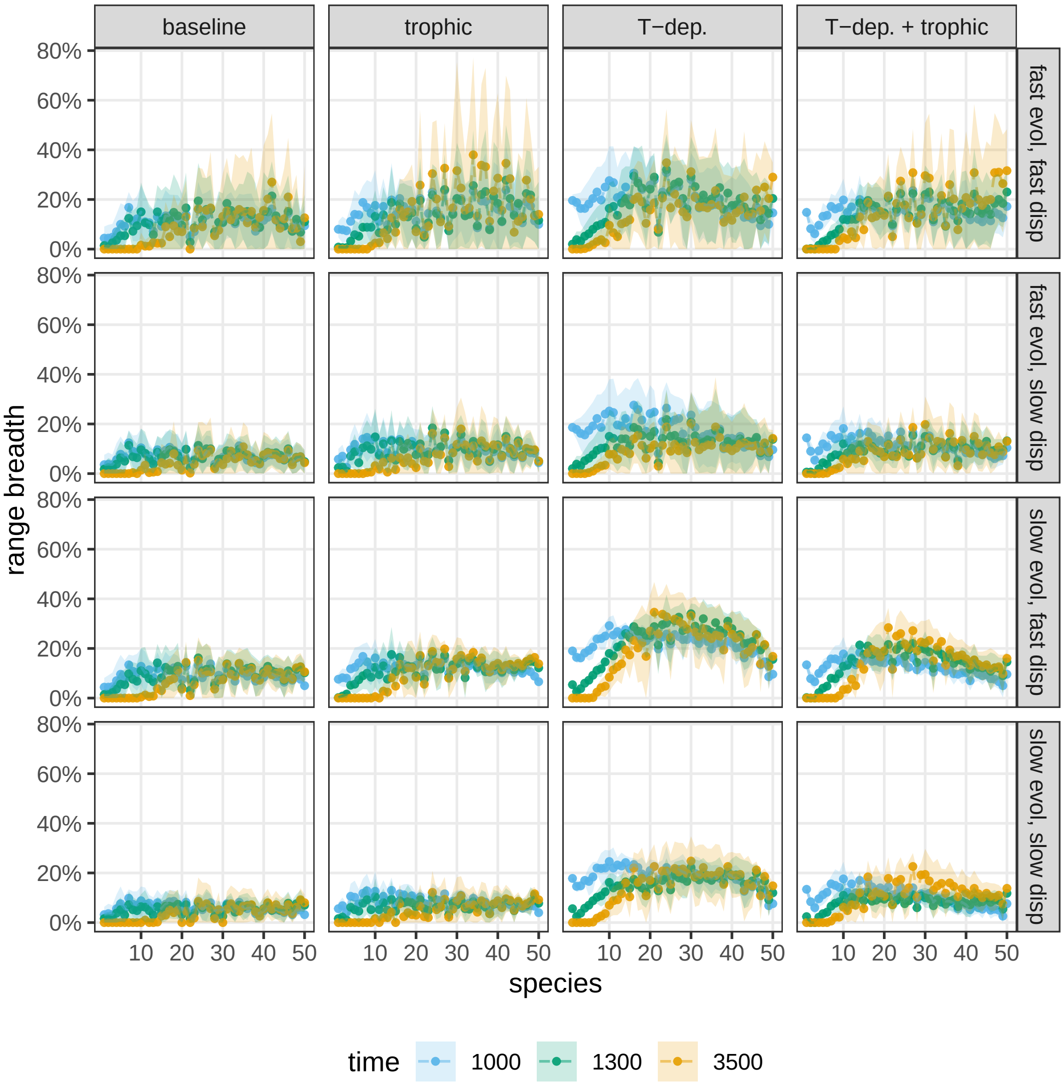

Supplementary Figure 12: As Figure 5 of the main text, but with generalist consumers.

5.8 Summary plots for consumers

Supplementary Figures 34-36 are like Figures 4-6 in the main text, except they show the distribution ranges, local diversity, and global diversity information for the consumer instead of the resource species. Supplementary Figure 37 is the analogue of Supplementary Figure 5 for consumers.

5.9 Results with 30 species per trophic level

Figures 4-6 in the main text assume 50 species per trophic level. This number is sufficient for all relevant community patterns to stabilize, such that adding more species has no more substantial effect. To show this, we display species ranges, local richness, and global richness for $S = 30$ species per trophic level (Supplementary Figures 38-46). The qualitative patterns are identical to those in Figures 4-7 of the main text, and Supplementary Figures 5 and 37.

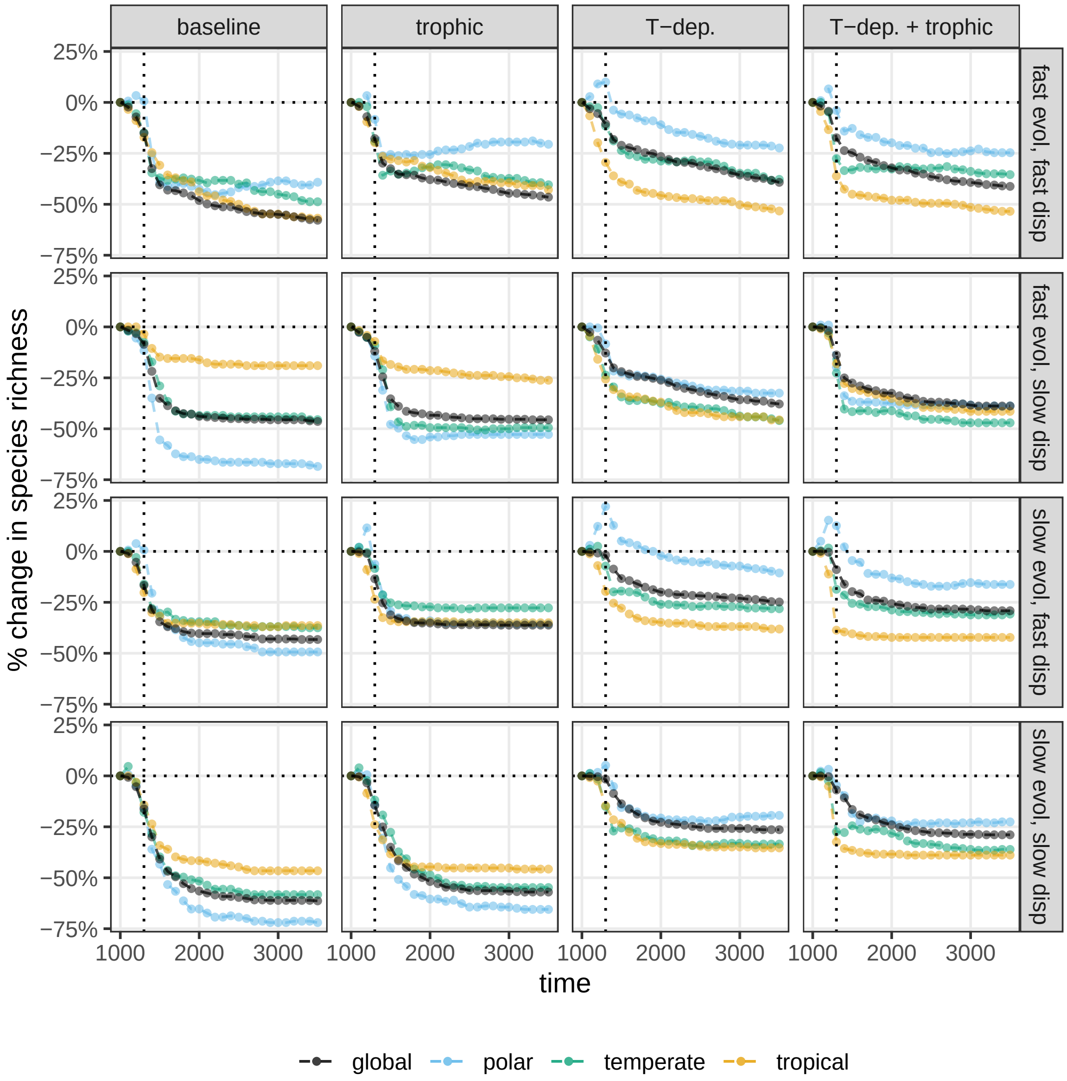

Supplementary Figure 13: As Figure 6 of the main text, but with generalist consumers.

5.10 Increasing the number of habitat patches from 50 to 100

Beyond a certain density of patches between pole and equator, results no longer change by adding even more patches. Our choice of $L = 50$ patches is sufficient for achieving this kind of convergence. To show this, Supplementary Figures 47, 48, and 49 below reproduce the main text's Figures 4, 5, and 6, respectively—but with 100 instead of 50 equally spaced patches. We generated only 20 instead of 100 replicates; nevertheless, the results are visibly unchanged by this doubling of the number of patches.

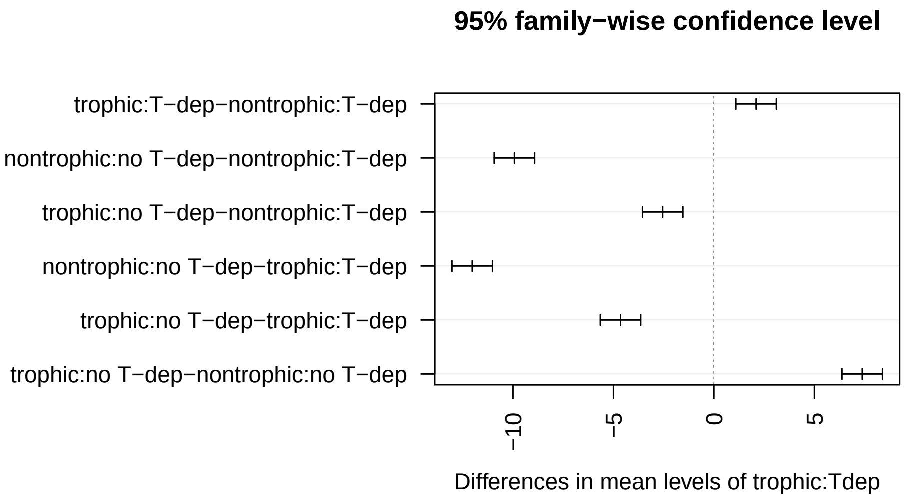

Supplementary Figure 14: Differences of the effect on local species diversity (abscissa) of different model setups (ordinate): models with- or without trophic interactions (trophic/nontrophic), and with- or without temperature-dependent competition (T-dep/no T-dep). Results shown for communities with high genetic variance and fast dispersal. As seen, the individual effects of trophic structure and temperature-dependent competition are both strong compared to the baseline model. However, their joint action does not create much of an effect above and beyond what they individually produce.

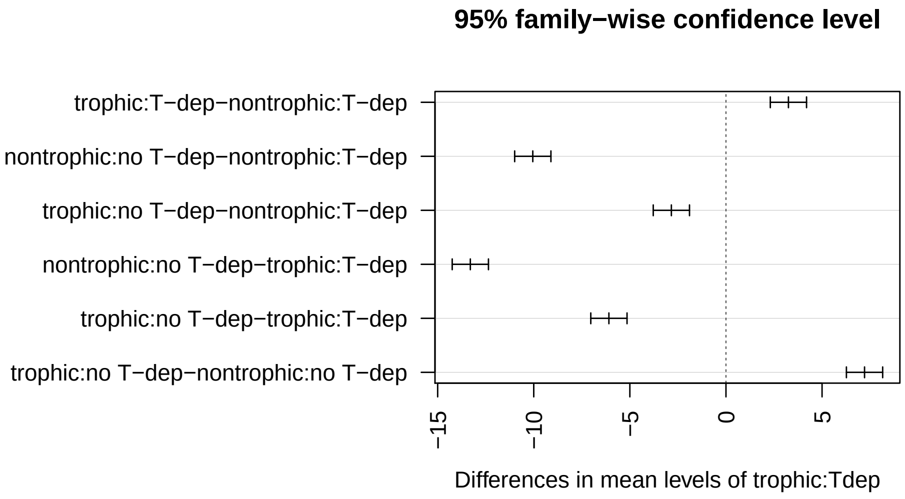

Supplementary Figure 15: As Supplementary Figure 14, but for communities with high genetic variance and slow dispersal.

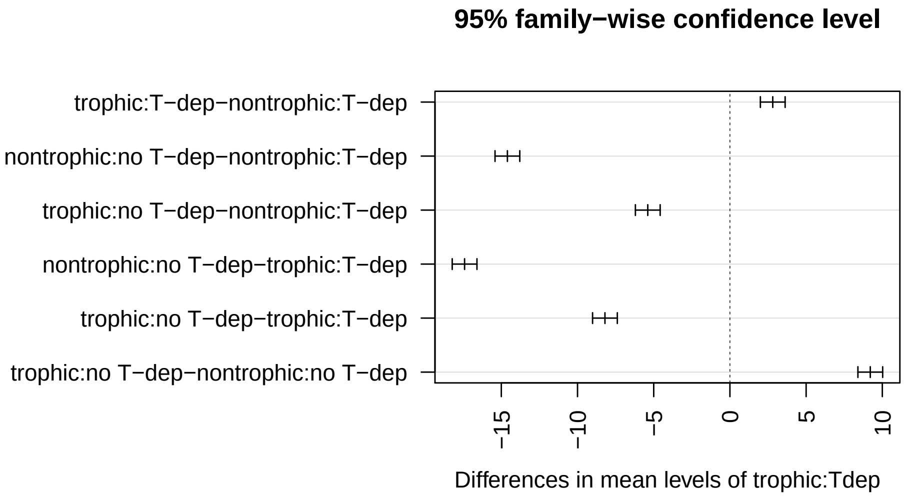

Supplementary Figure 16: As Supplementary Figure 14, but for communities with low genetic variance and fast dispersal.

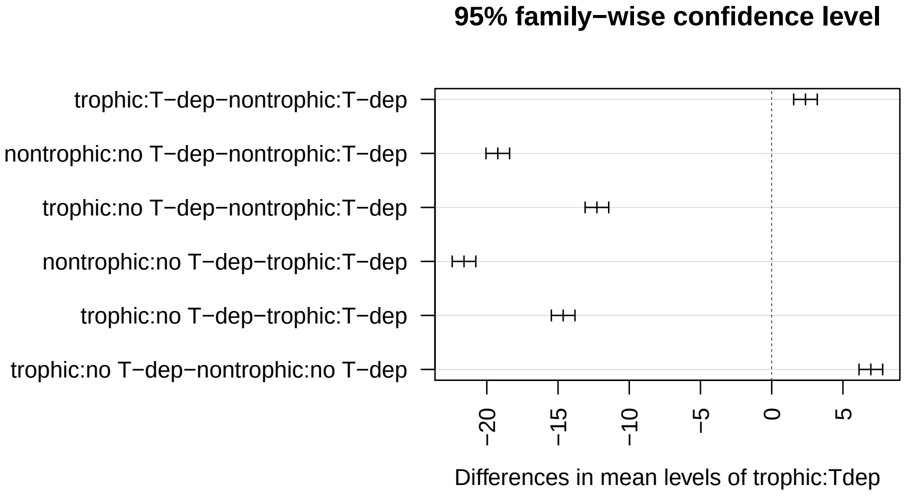

Supplementary Figure 17: As Supplementary Figure 14, but for communities with low genetic variance and slow dispersal.

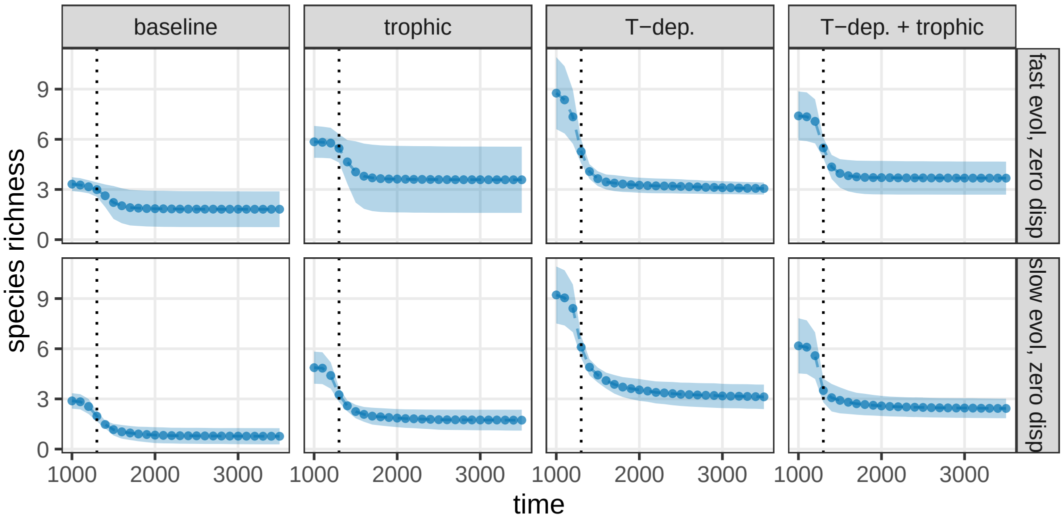

Supplementary Figure 18: As Figure 4 of the main text, but with dispersal set to zero.

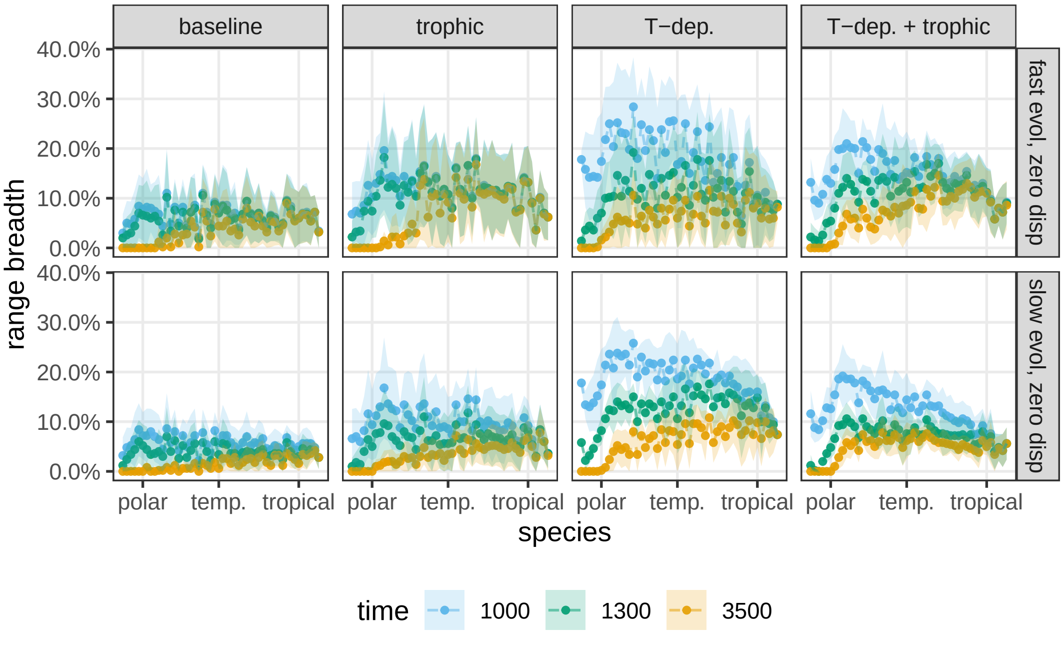

Supplementary Figure 19: As Figure 5 of the main text, but with dispersal set to zero.

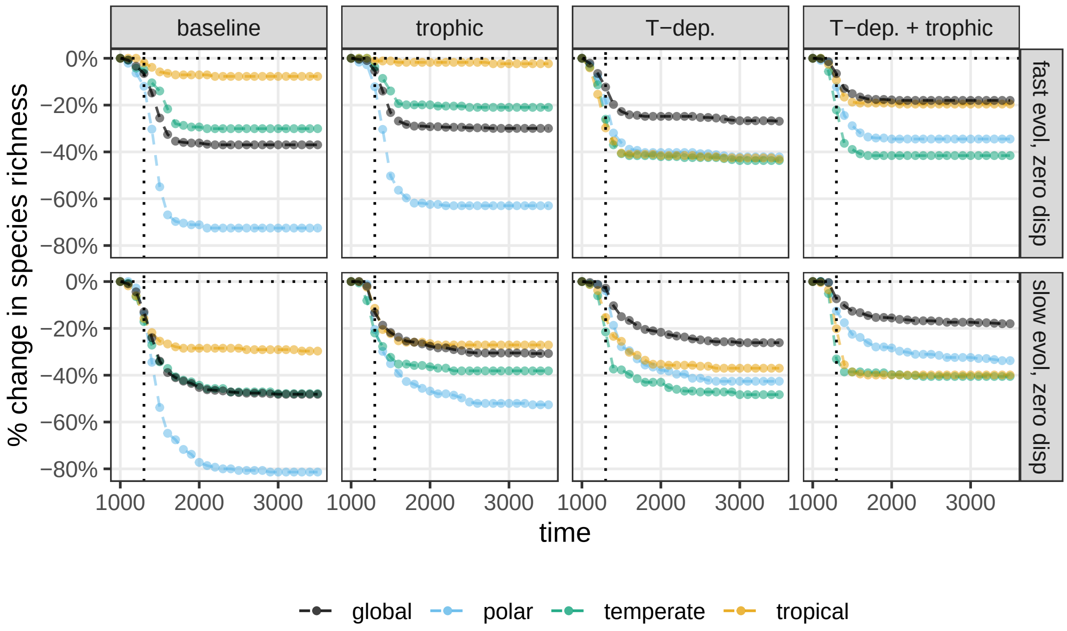

Supplementary Figure 20: As Figure 6 of the main text, but with dispersal set to zero.

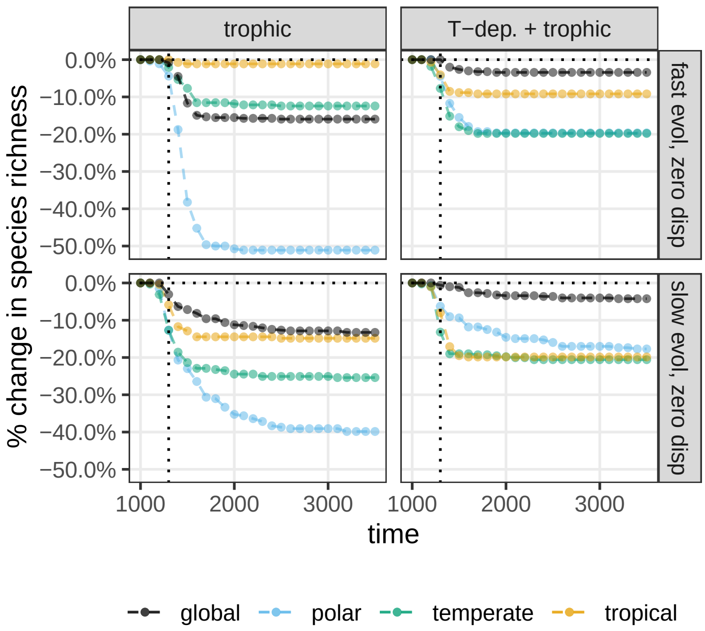

Supplementary Figure 21: As Figure 6 of the main text, but with dispersal set to zero, and for consumer species instead of resources.

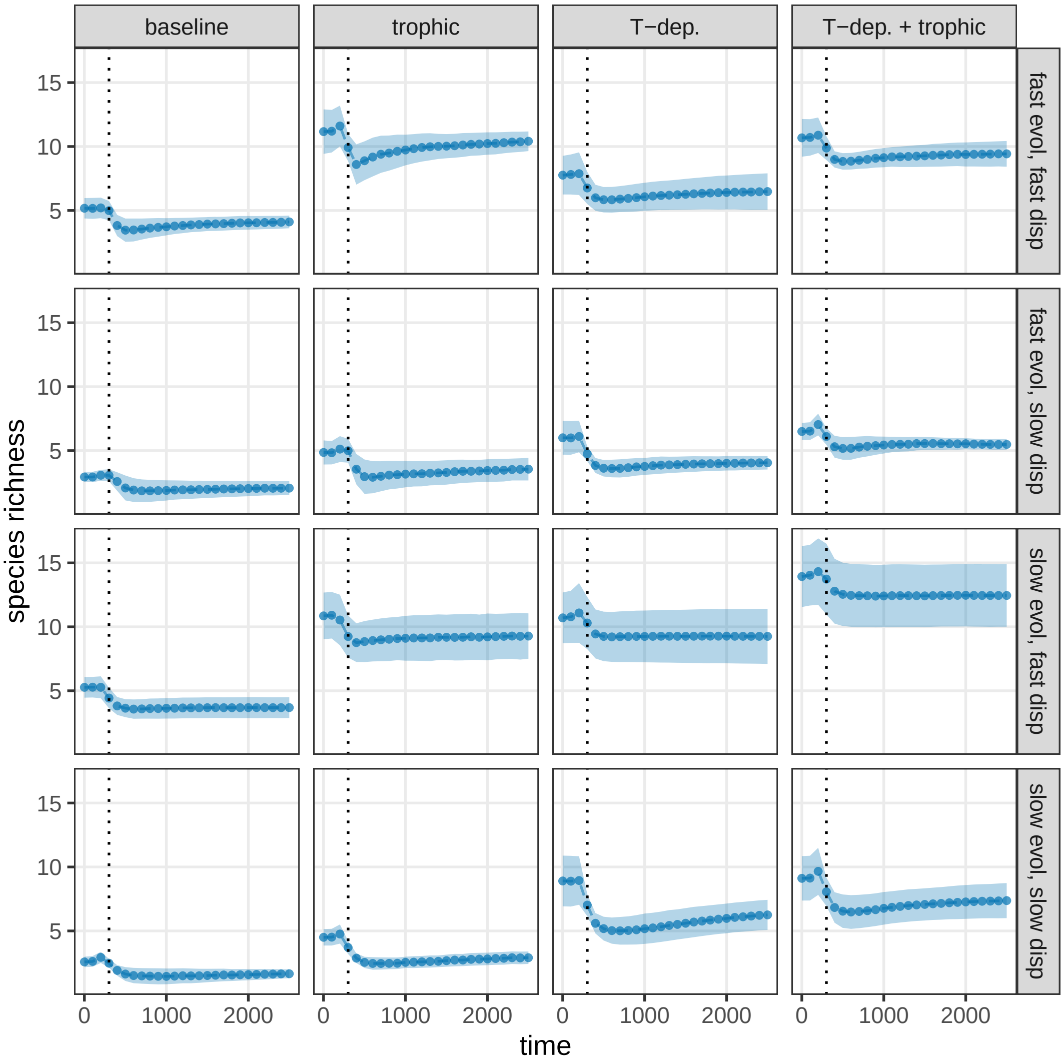

Supplementary Figure 22: As Figure 4 in the main text, but with a Gaussian dispersal kernel.

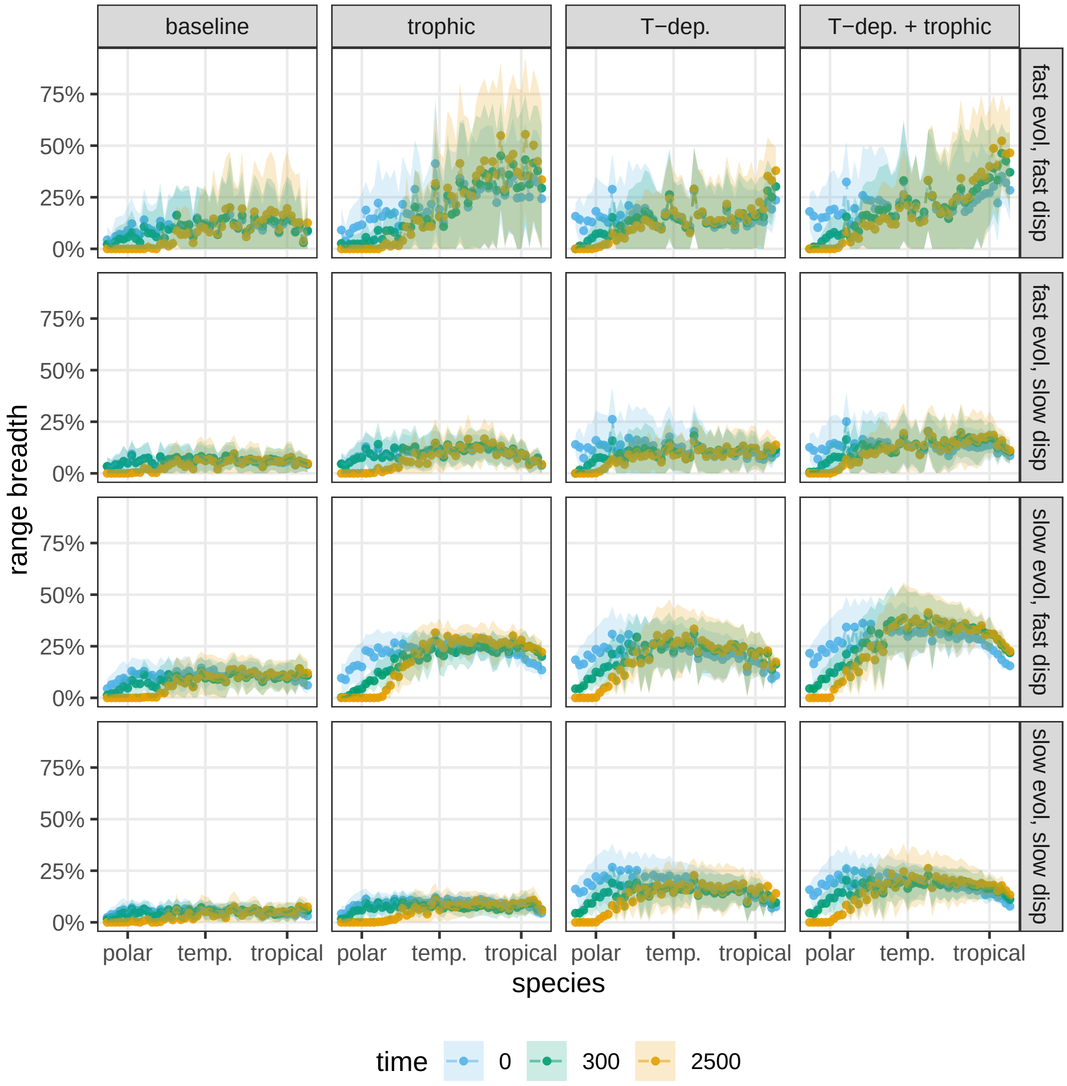

Supplementary Figure 23: As Figure 5 in the main text, but with a Gaussian dispersal kernel.

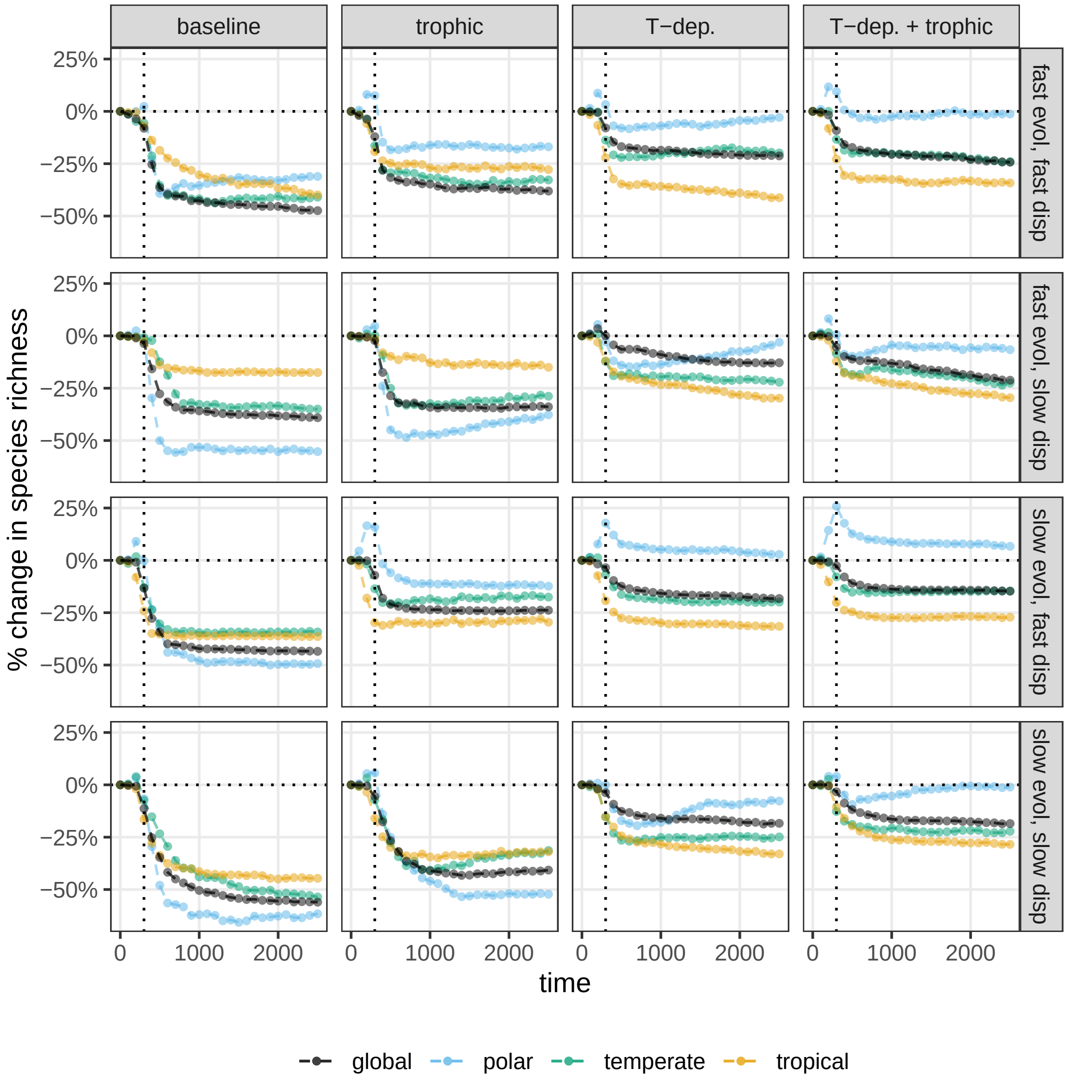

Supplementary Figure 24: As Figure 6 in the main text, but with a Gaussian dispersal kernel.

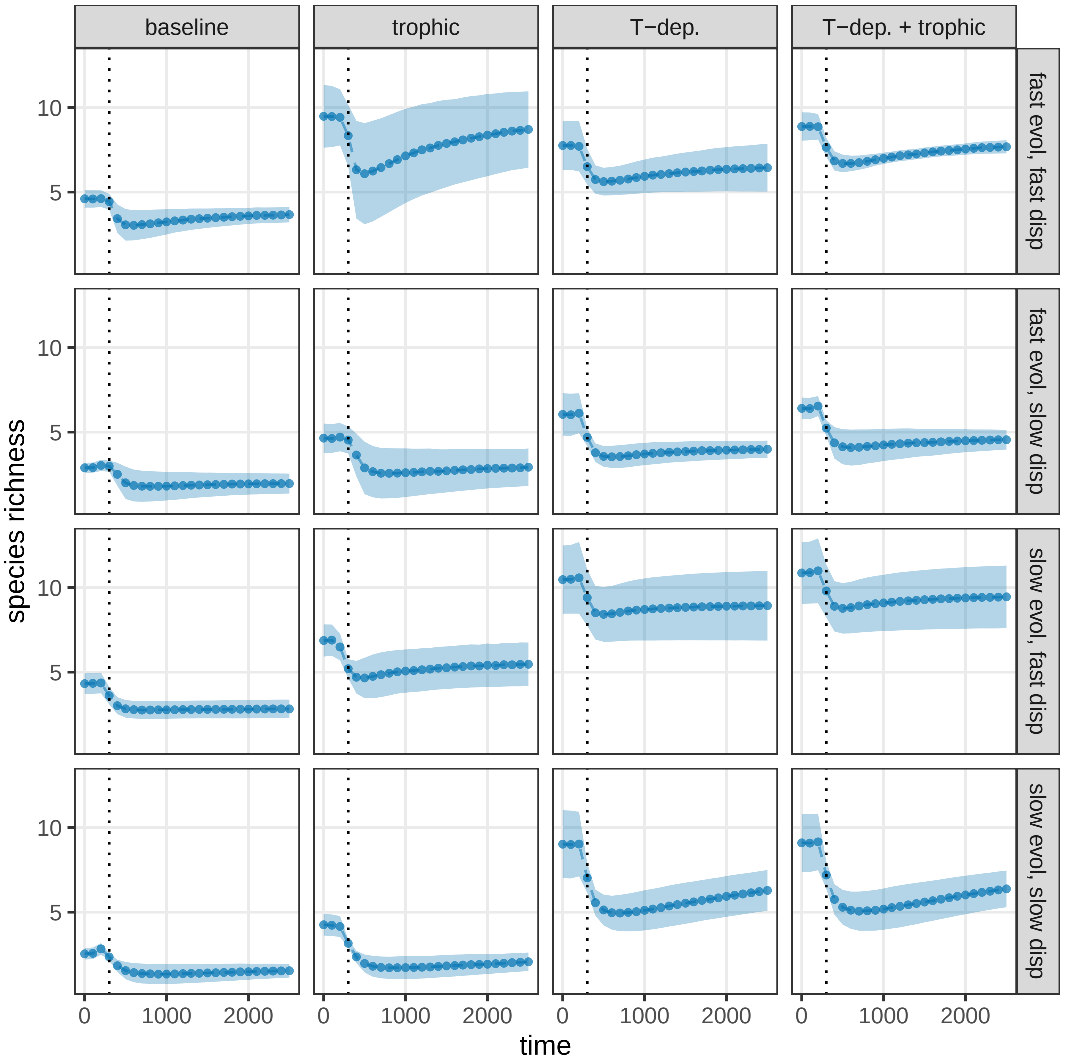

Supplementary Figure 25: As Figure 4 in the main text, but with an asymmetric rectangular dispersal kernel, northern bias.

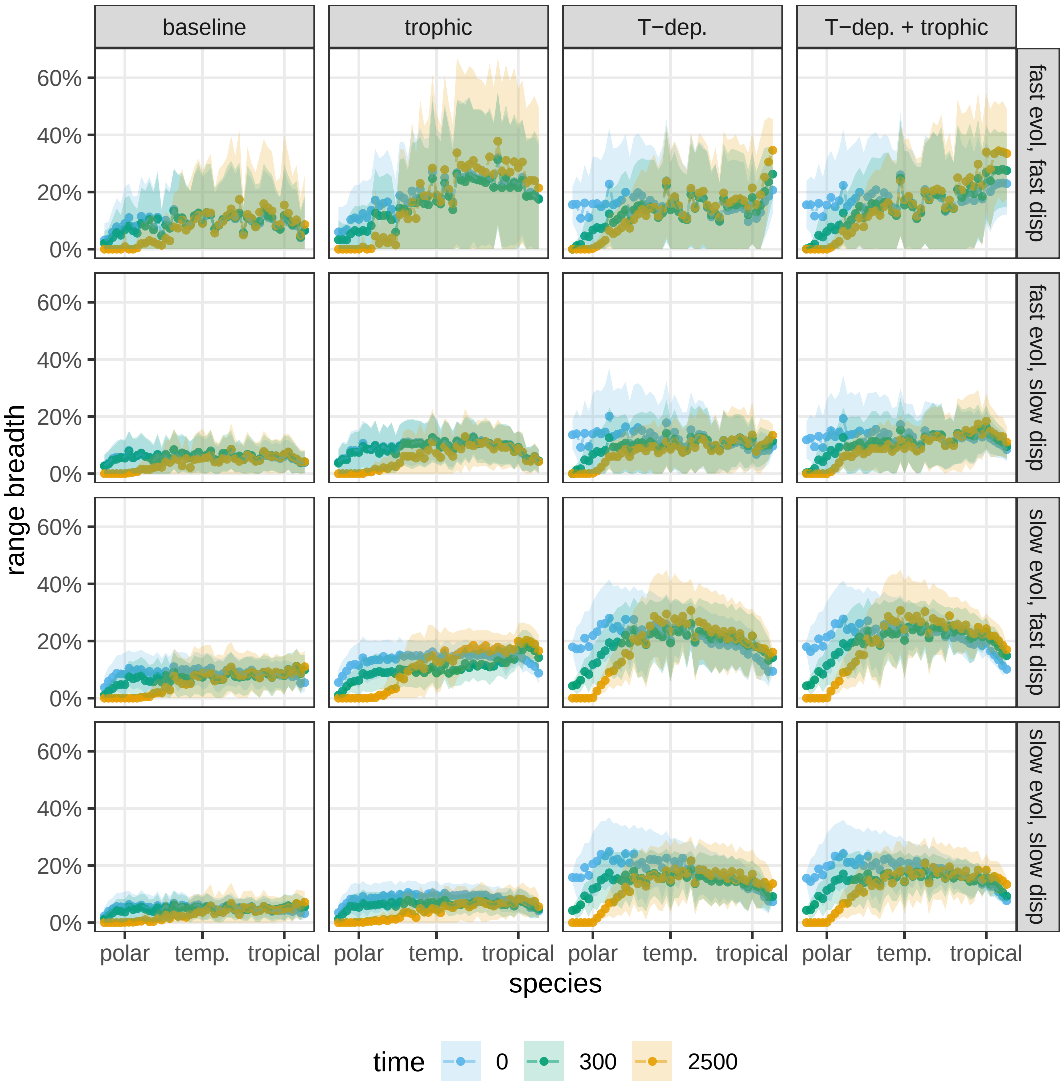

Supplementary Figure 26: As Figure 5 in the main text, but with an asymmetric rectangular dispersal kernel, northern bias.

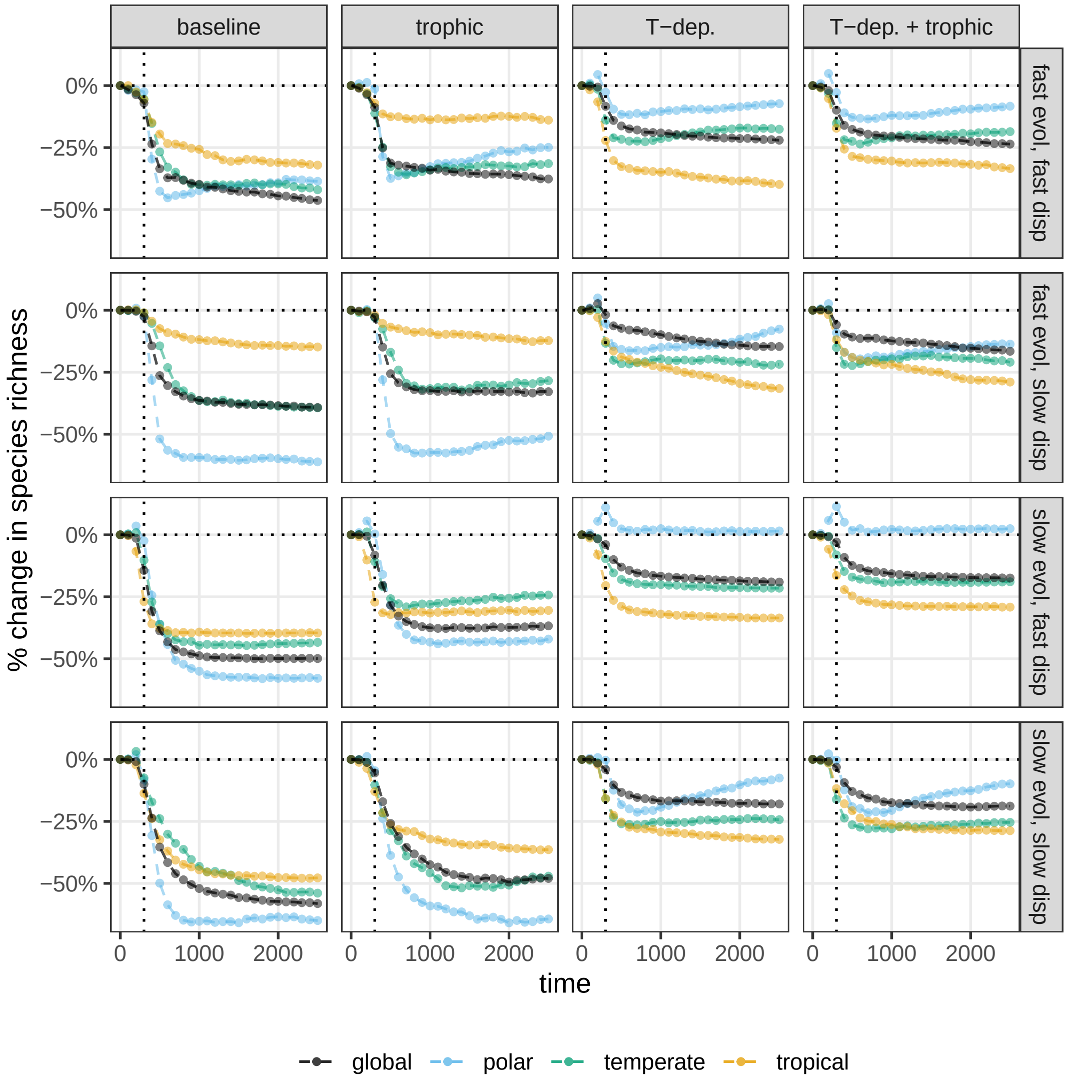

Supplementary Figure 27: As Figure 6 in the main text, but with an asymmetric rectangular dispersal kernel, northern bias.

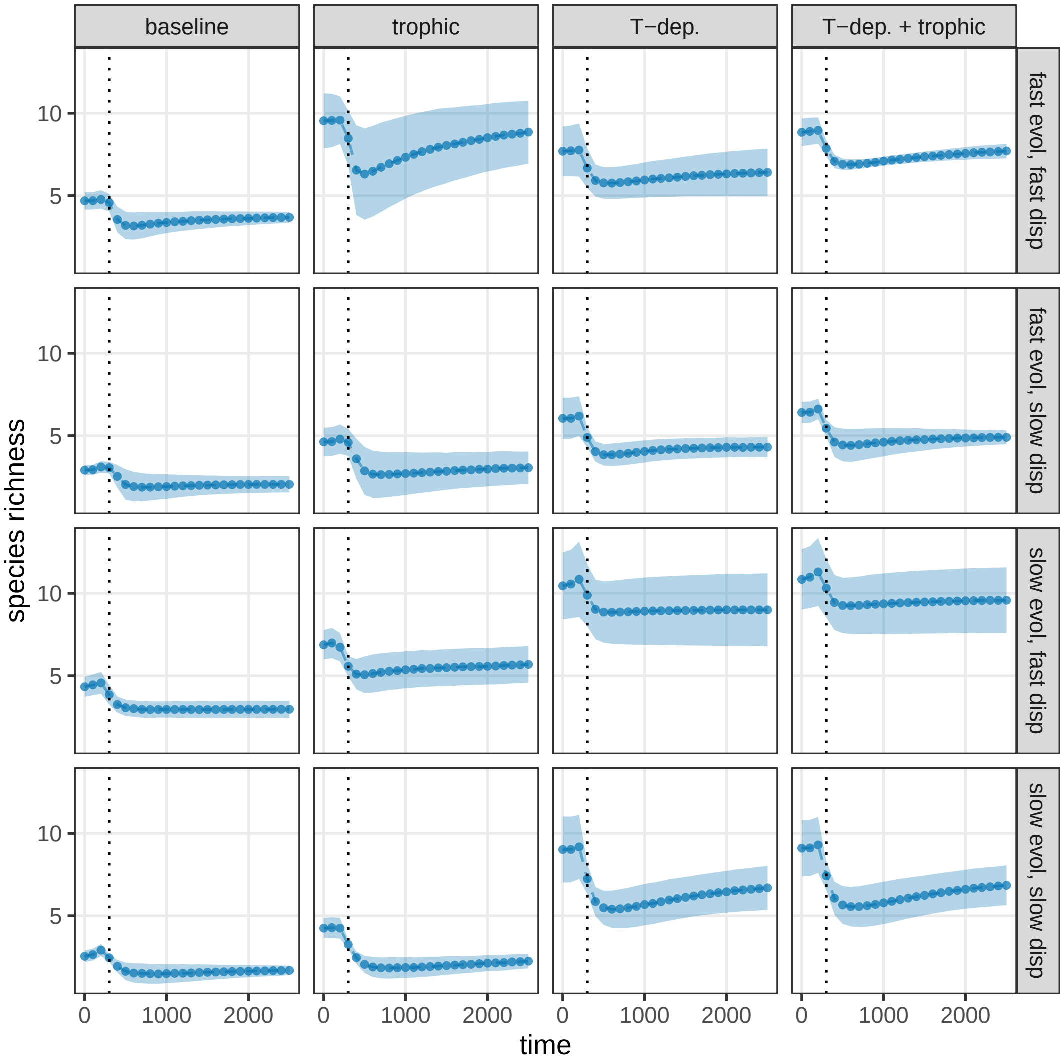

Supplementary Figure 28: As Figure 4 in the main text, but with an asymmetric rectangular dispersal kernel, southern bias.

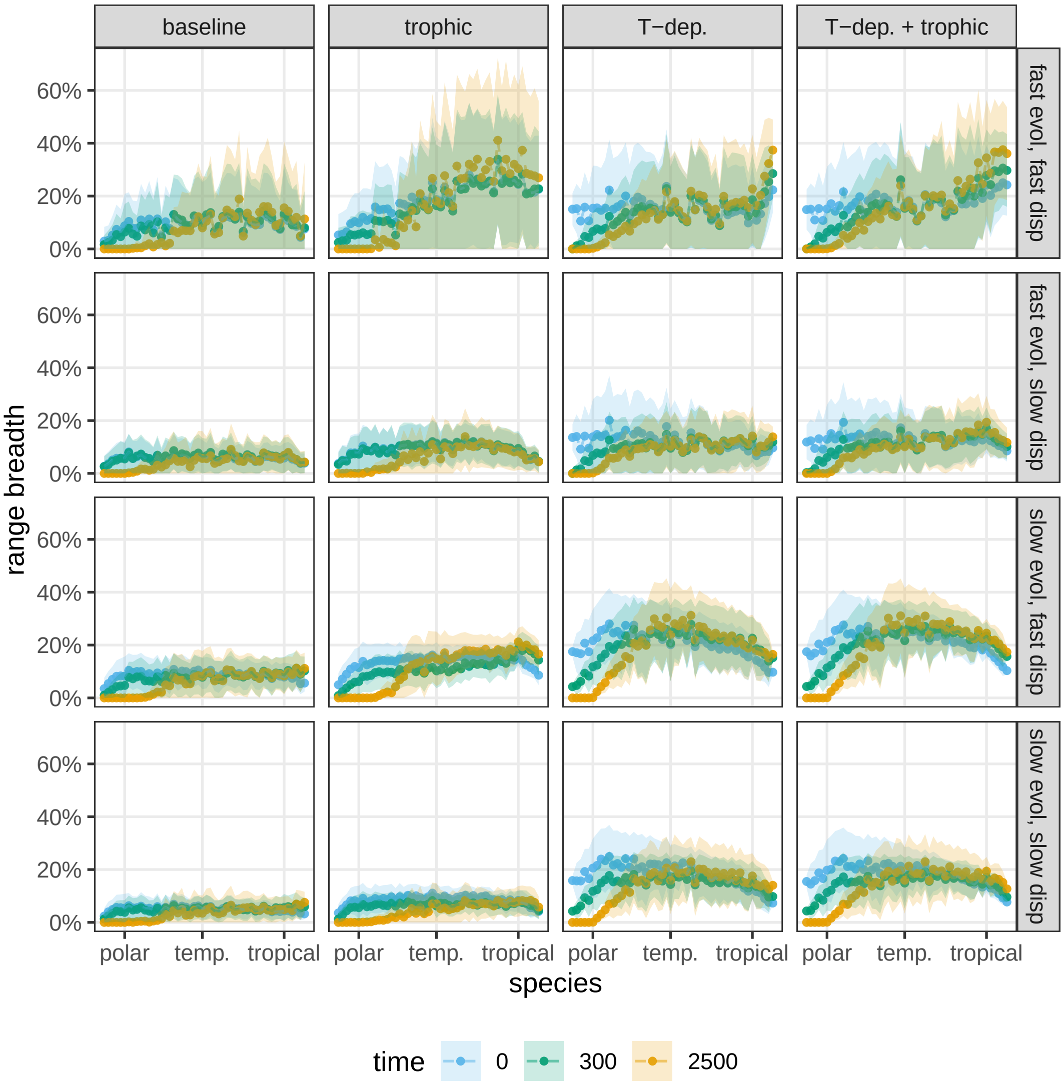

Supplementary Figure 29: As Figure 5 in the main text, but with an asymmetric rectangular dispersal kernel, southern bias.

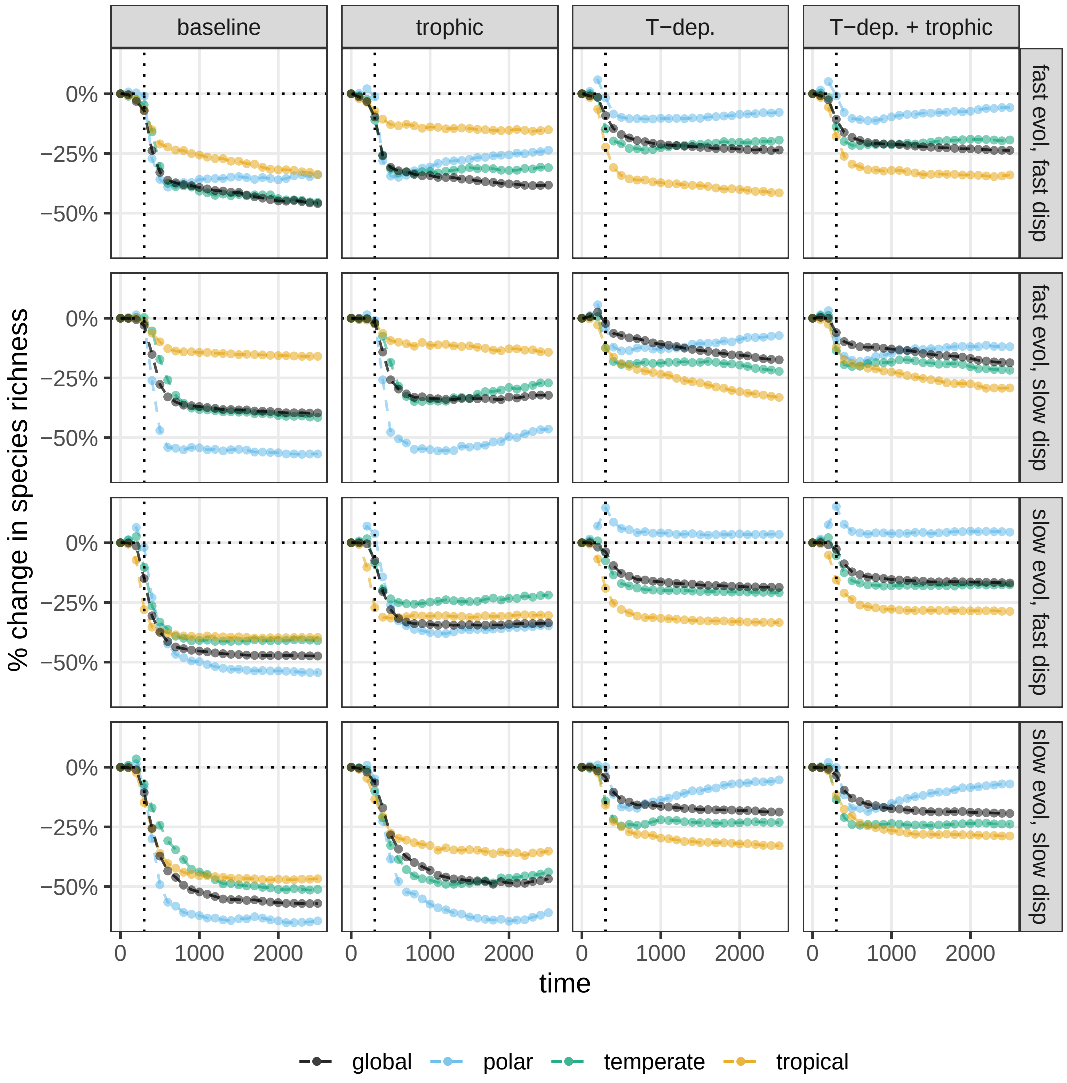

Supplementary Figure 30: As Figure 6 in the main text, but with an asymmetric rectangular dispersal kernel, southern bias.

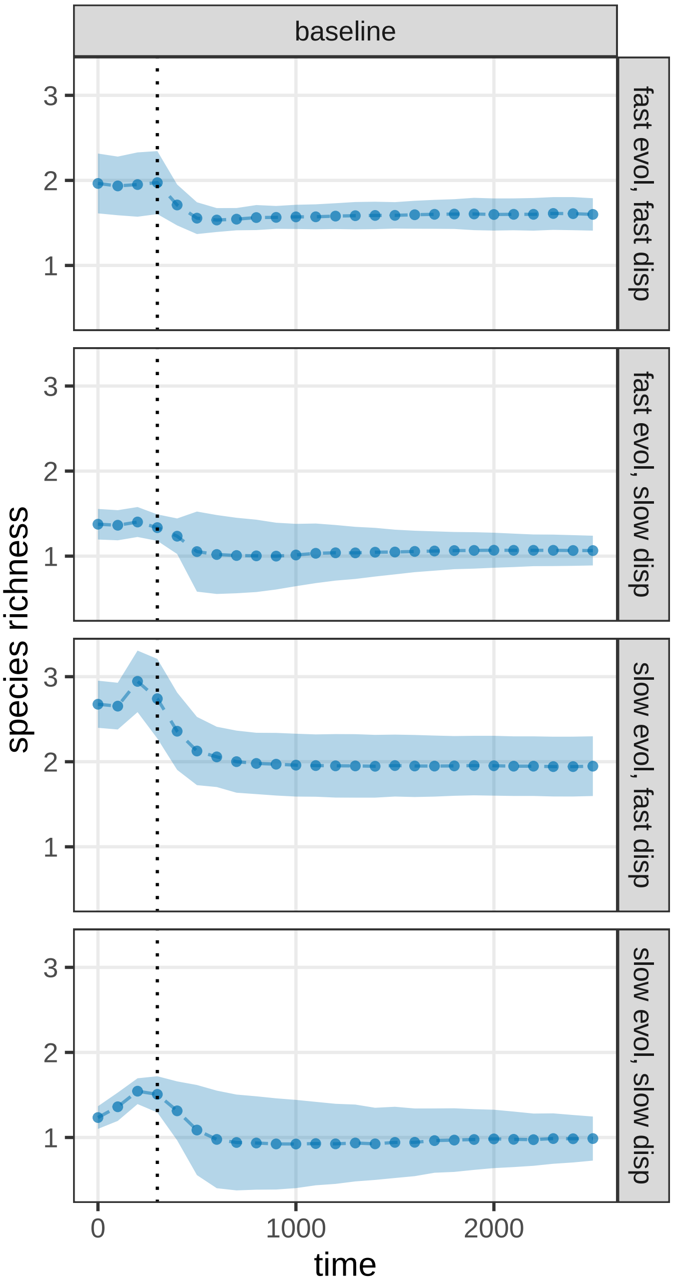

Supplementary Figure 31: As Figure 4 of the main text, but with resource species' intra- and interspecific competition coefficients set equal.

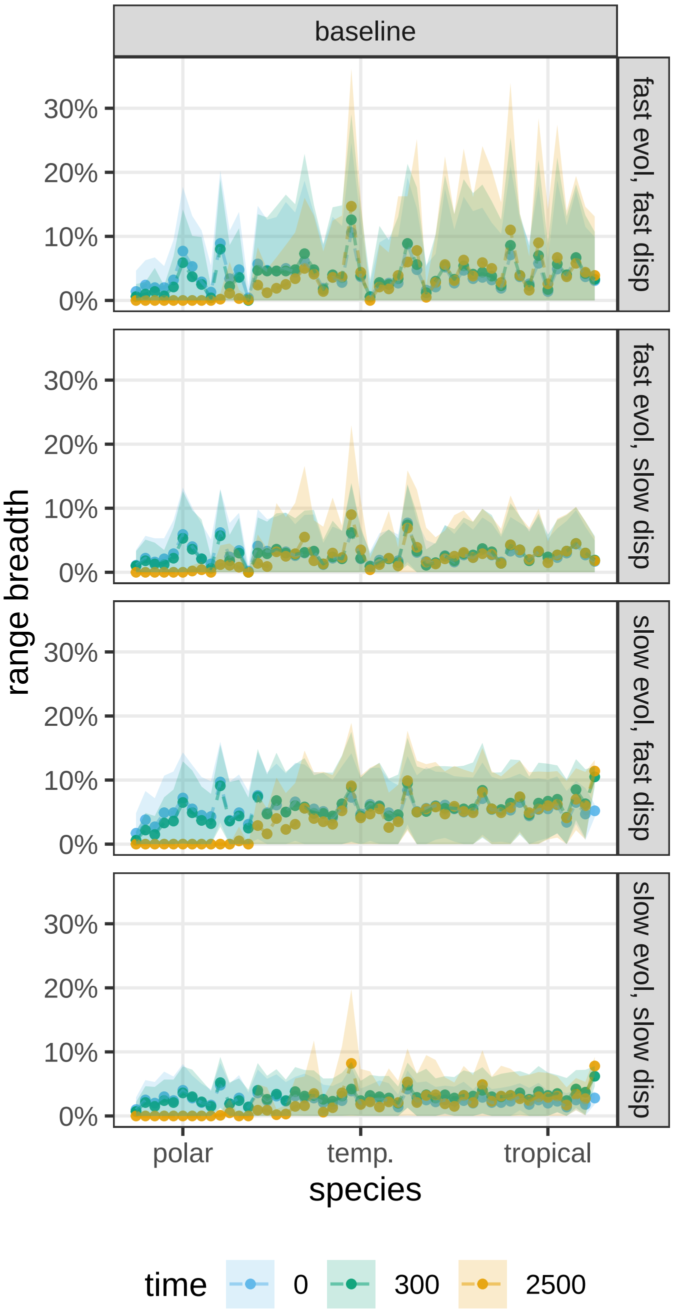

Supplementary Figure 32: As Figure 5 of the main text, but with resource species' intra- and interspecific competition coefficients set equal.

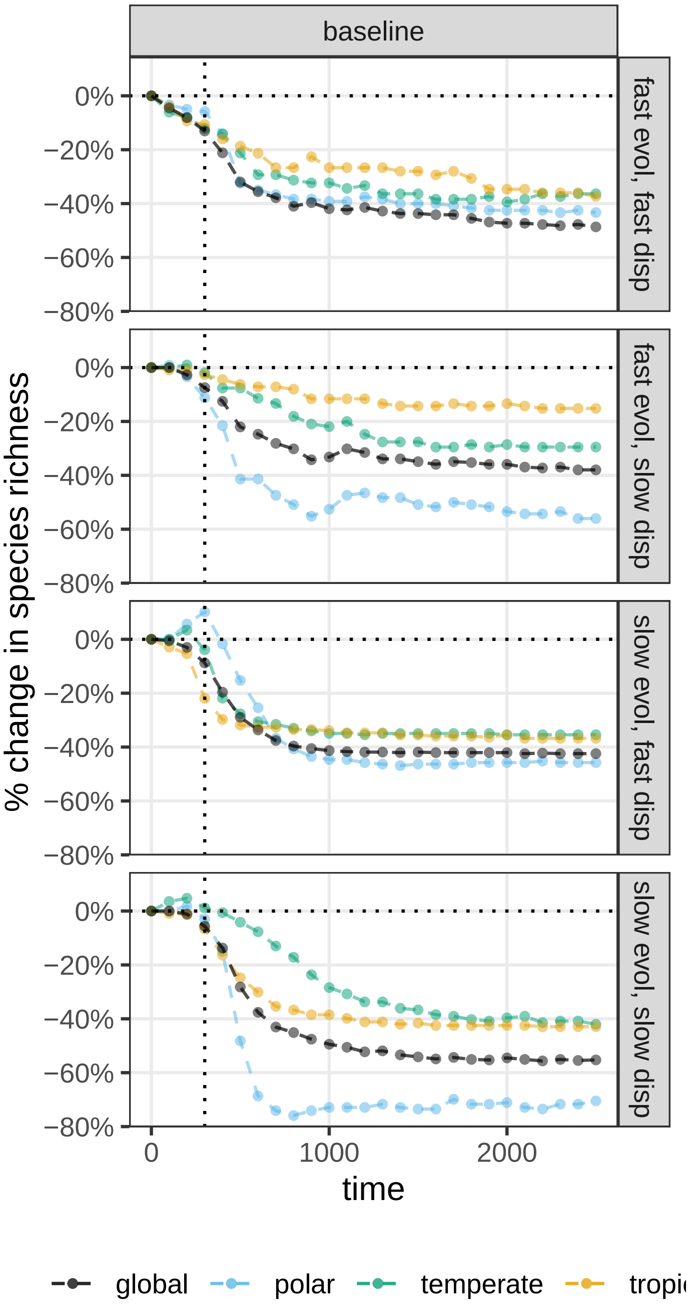

Supplementary Figure 33: As Figure 6 of the main text, but with resource species' intra- and interspecific competition coefficients set equal.

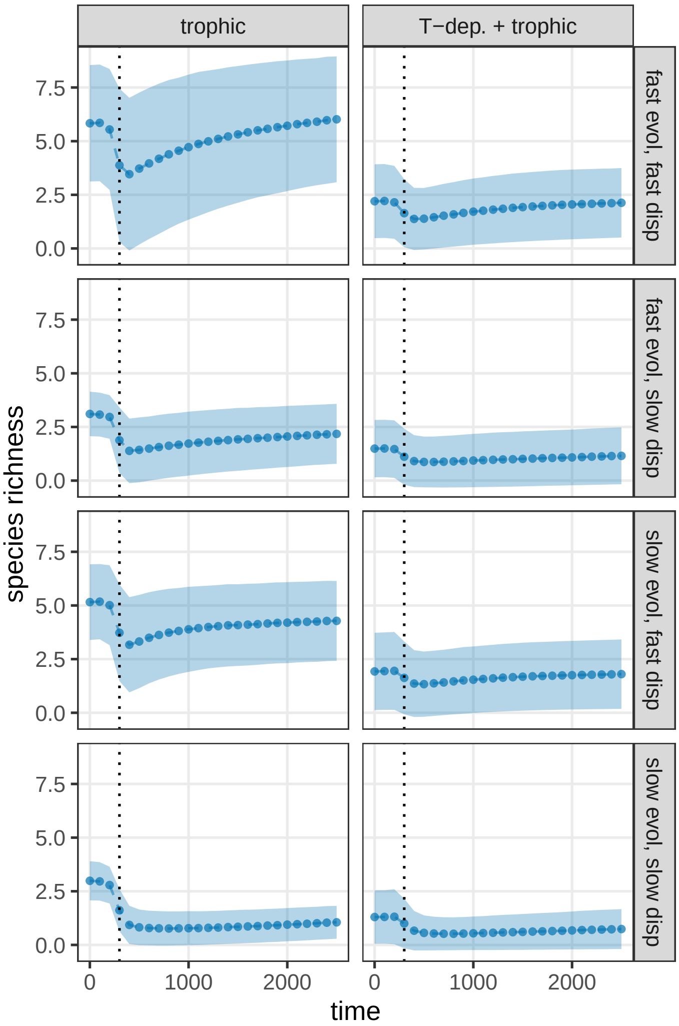

Supplementary Figure 34: As Figure 4 of the main text, but for consumer species (species 51 to 100).

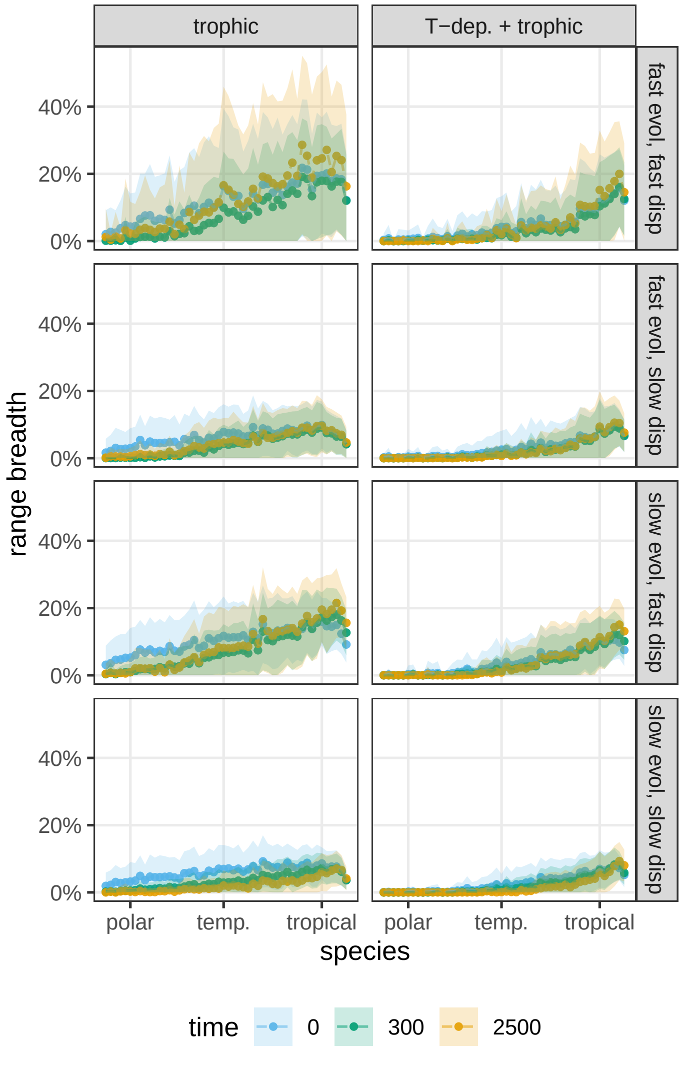

Supplementary Figure 35: As Figure 5 of the main text, but for consumer species (species 51 to 100).

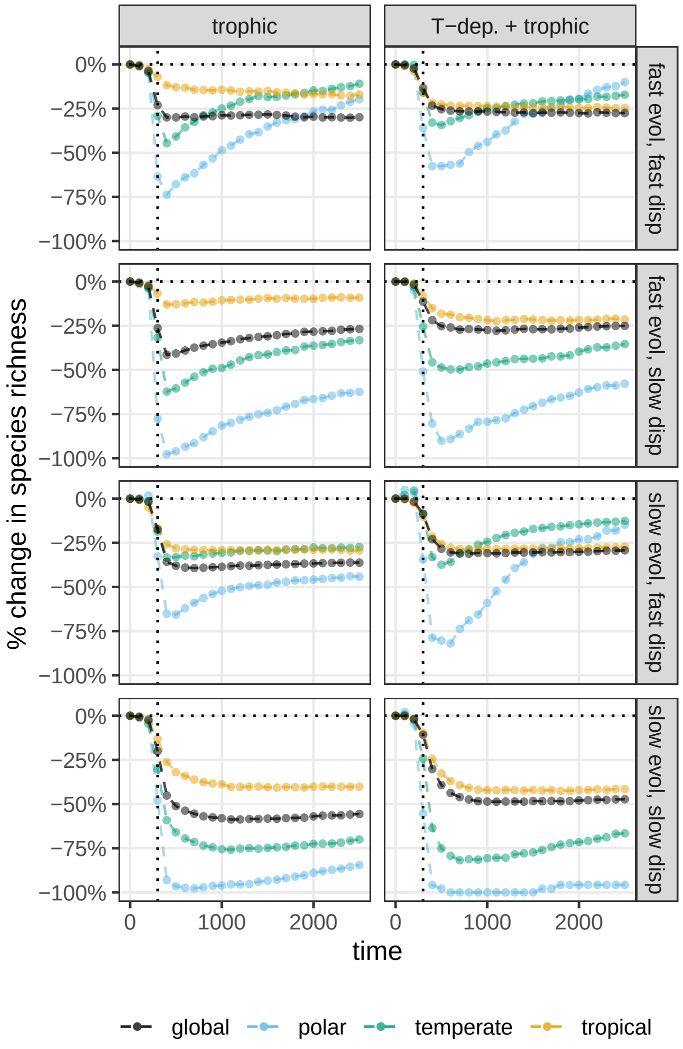

Supplementary Figure 36: As Figure 6 of the main text, but for consumer species (species 51 to 100).

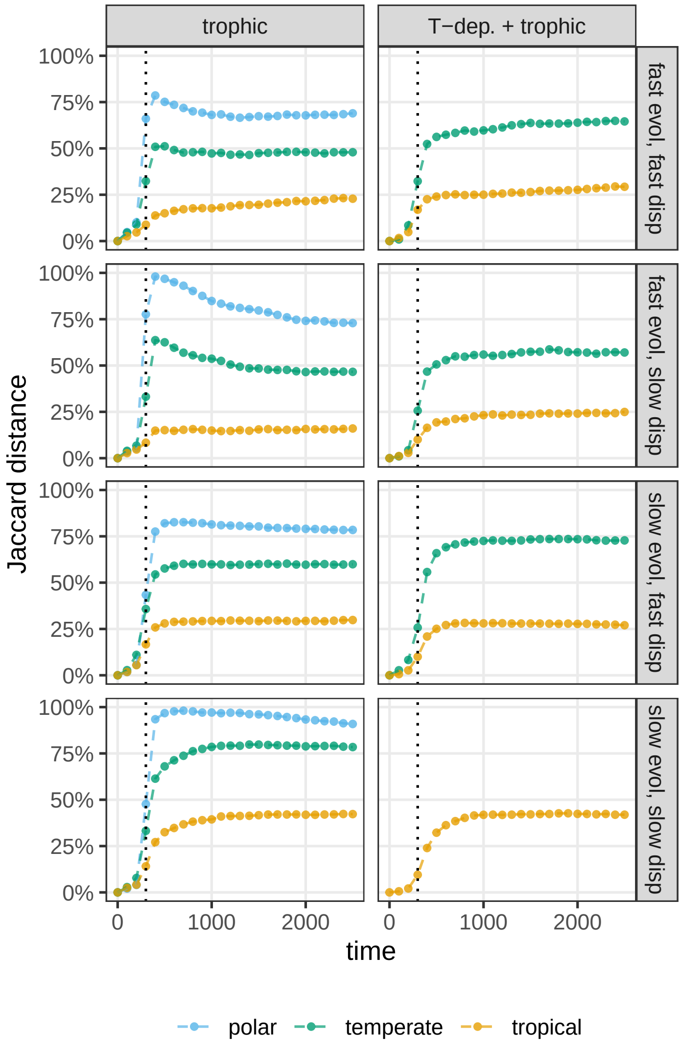

Supplementary Figure 37: As Supplementary Figure 5, but for consumer species (species 51 to 100).

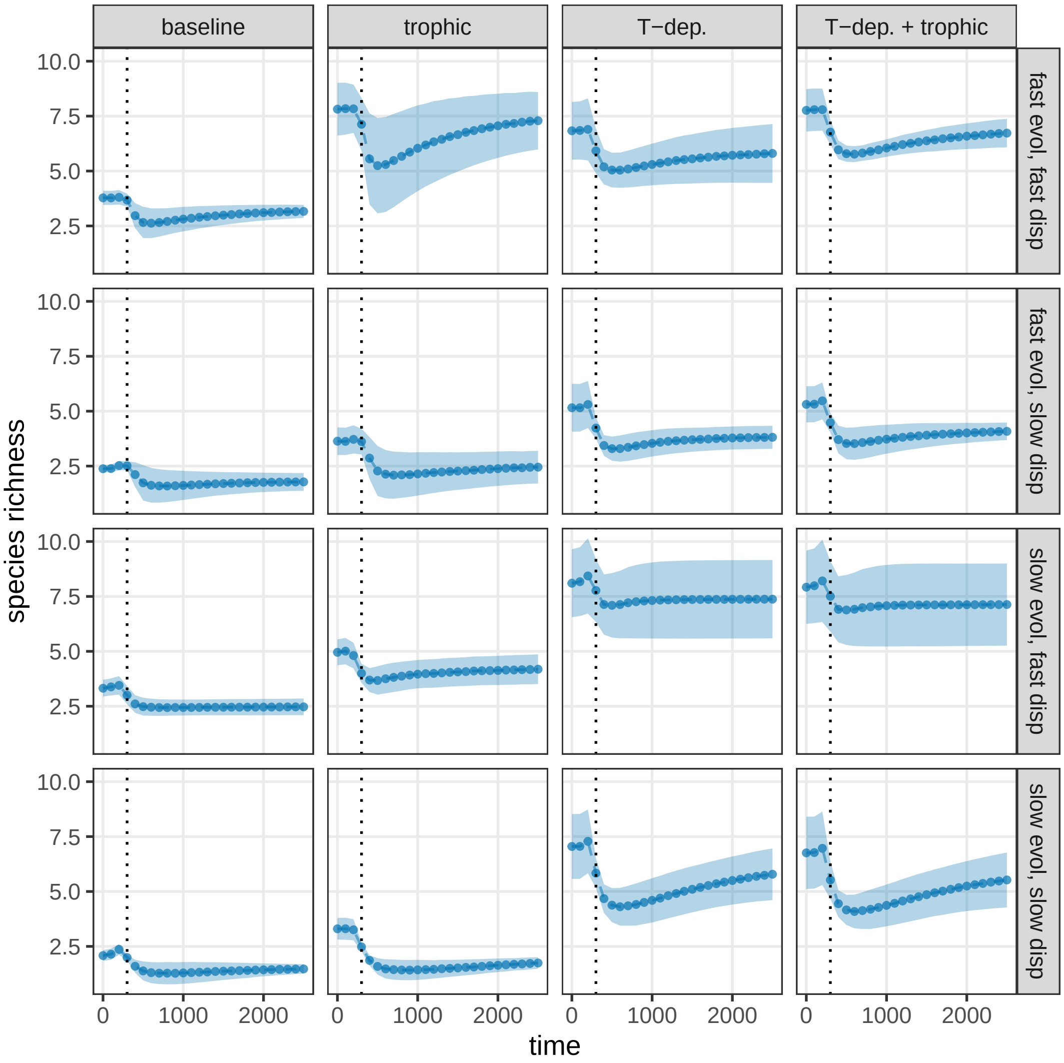

Supplementary Figure 38: As Figure 4 of the main text, but with $S = 30$ resource species.

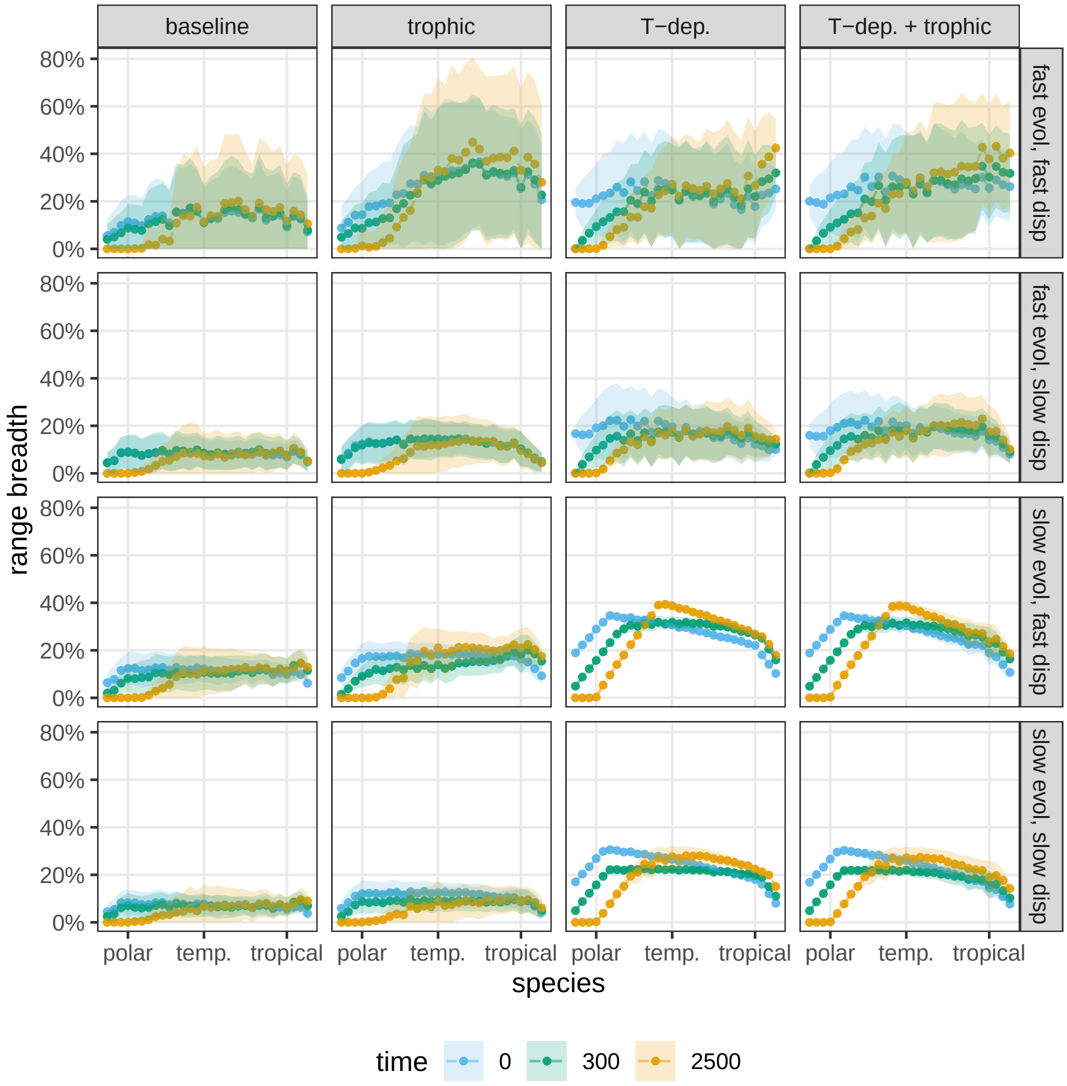

Supplementary Figure 39: As Figure 5 of the main text, but with $S = 30$ resource species.

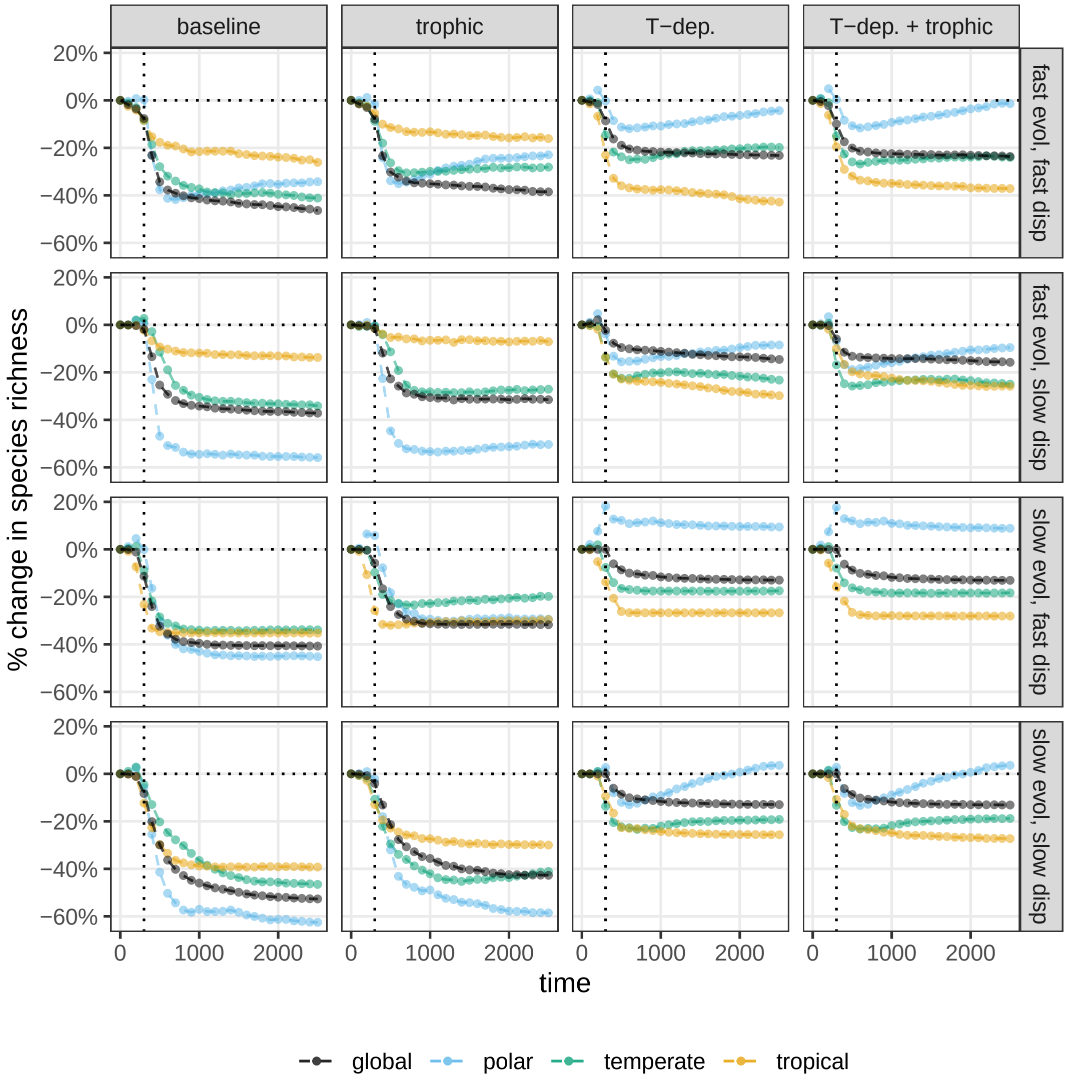

Supplementary Figure 40: As Figure 6 of the main text, but with $S = 30$ resource species.

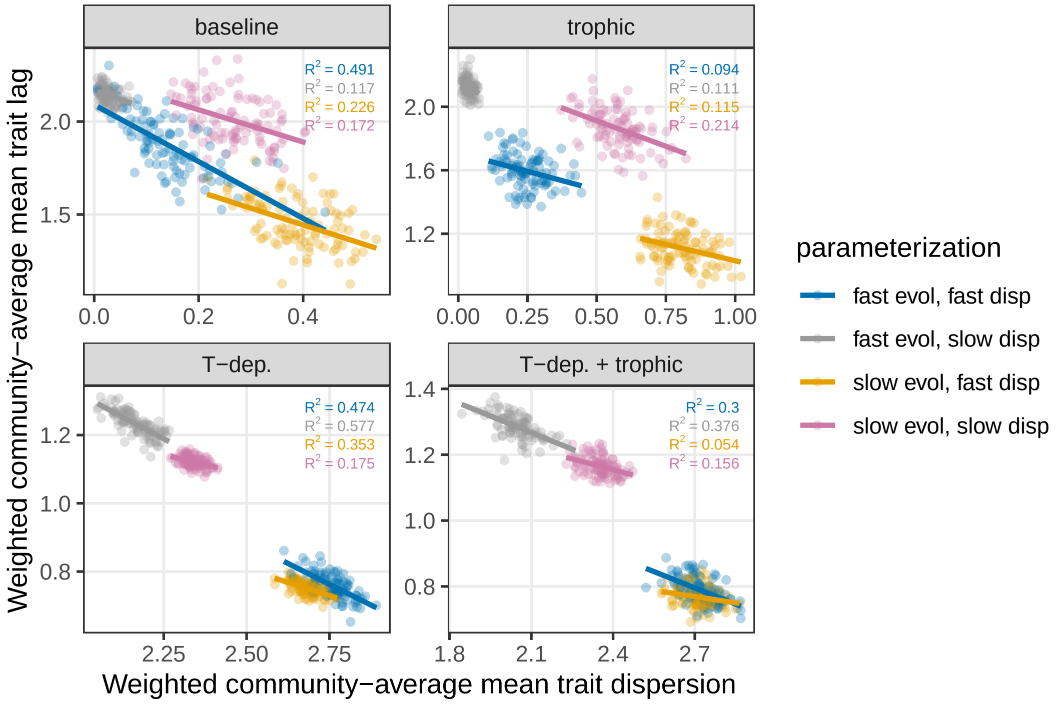

Supplementary Figure 41: As Figure 7 in the main text, but with $S = 30$ resource species.

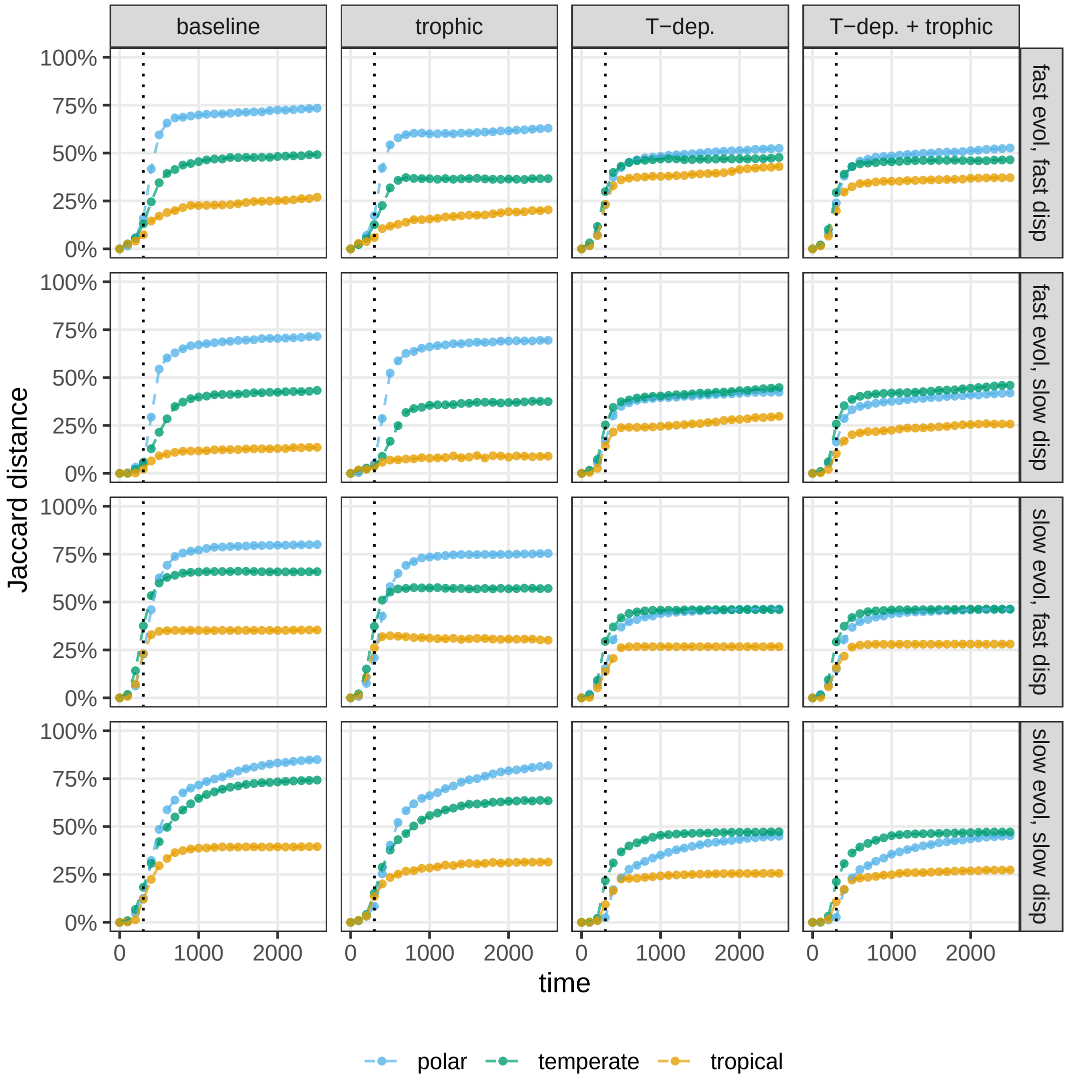

Supplementary Figure 42: As Supplementary Figure 5, but with $S = 30$ resource species.

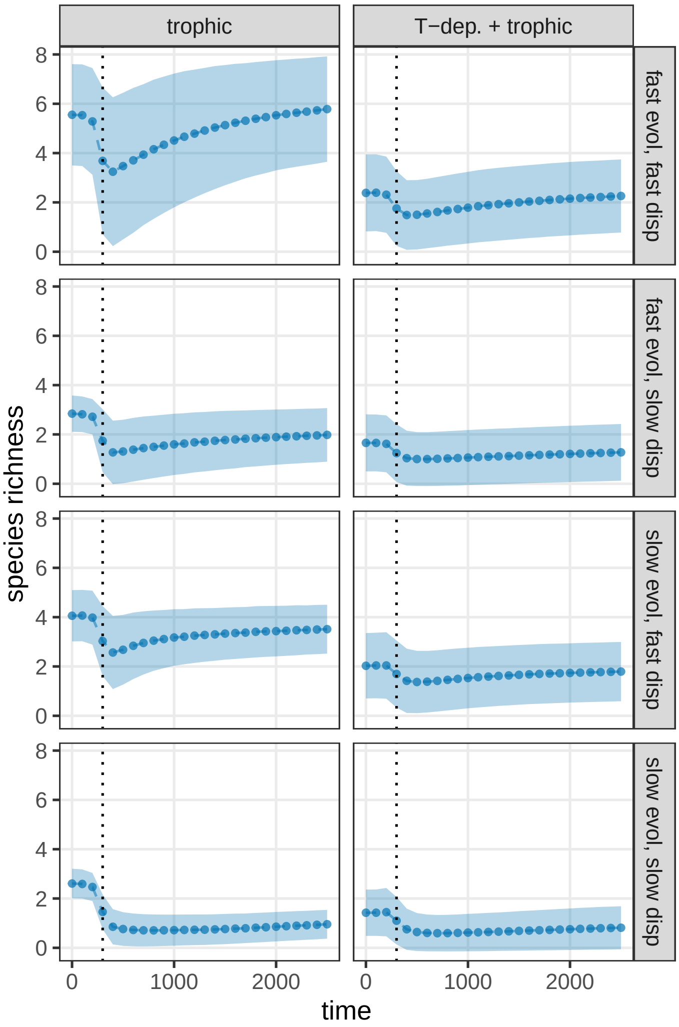

Supplementary Figure 43: As Supplementary Figure 34, but with $S = 30$ consumer species (species 31 to 60).

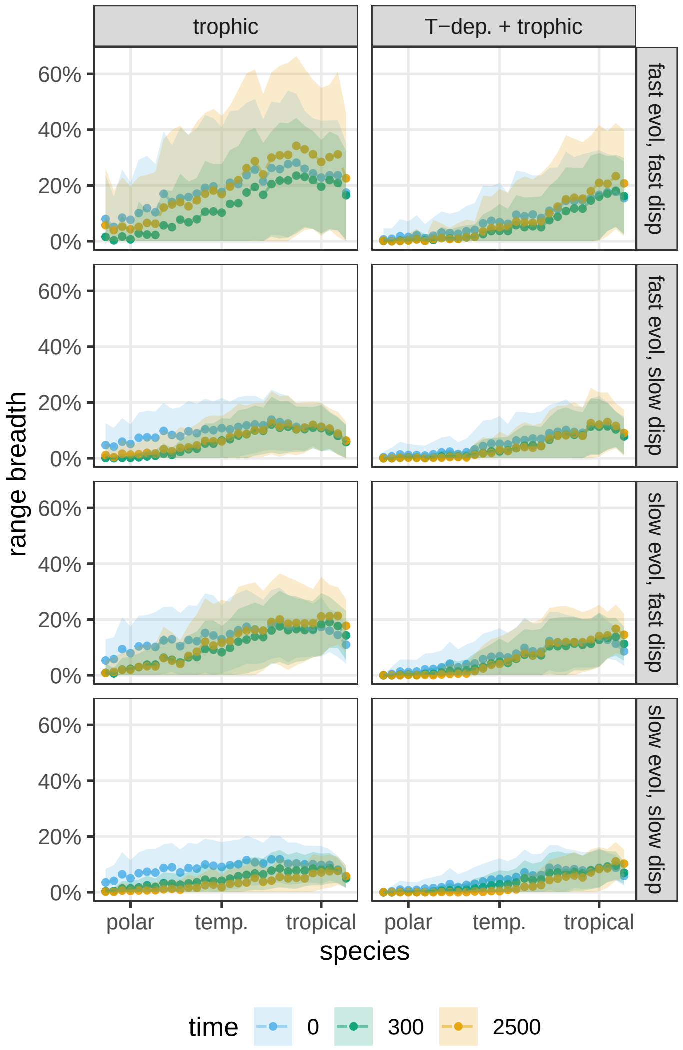

Supplementary Figure 44: As Supplementary Figure 35, but with $S = 30$ consumer species (species 31 to 60).

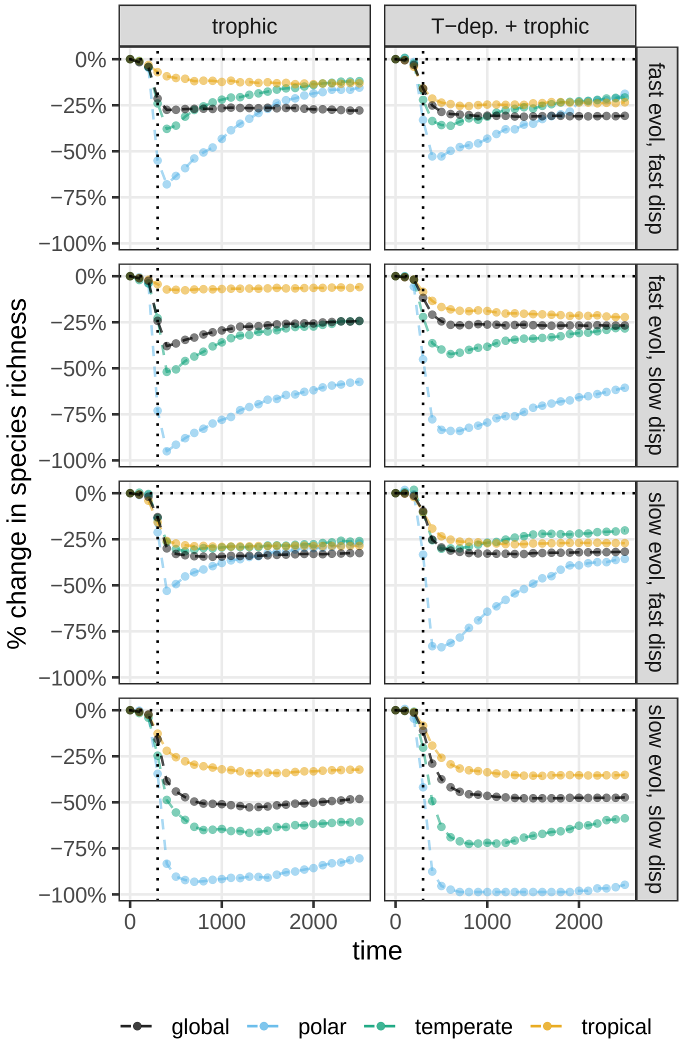

Supplementary Figure 45: As Supplementary Figure 36, but with $S = 30$ consumer species (species 31 to 60).

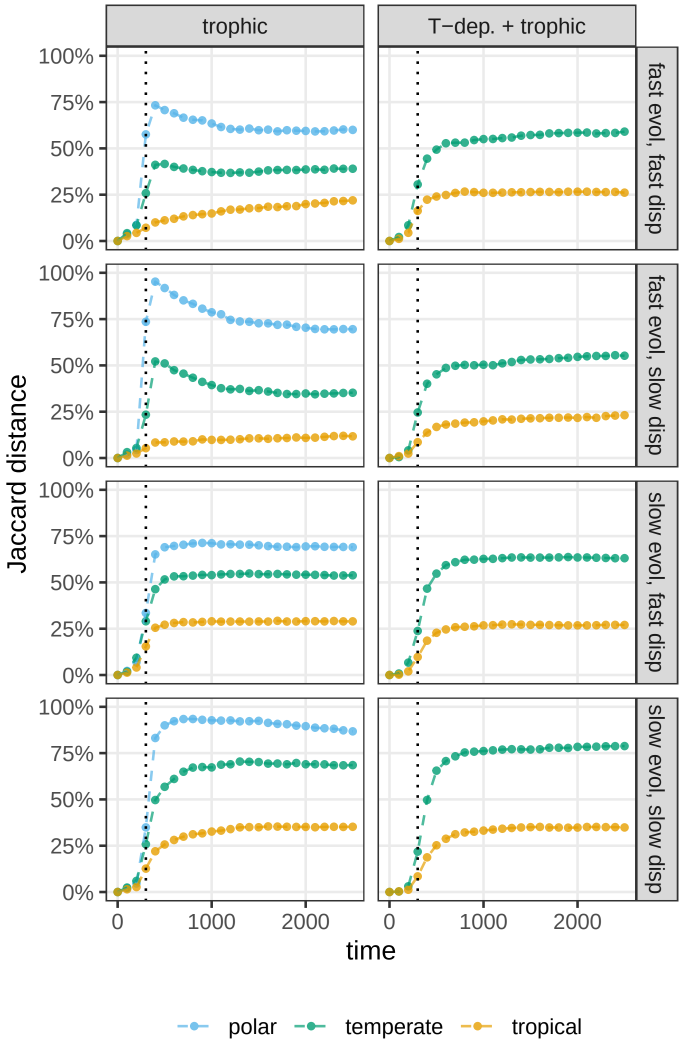

Supplementary Figure 46: As Supplementary Figure 37, but with $S = 30$ consumer species (species 31 to 60).

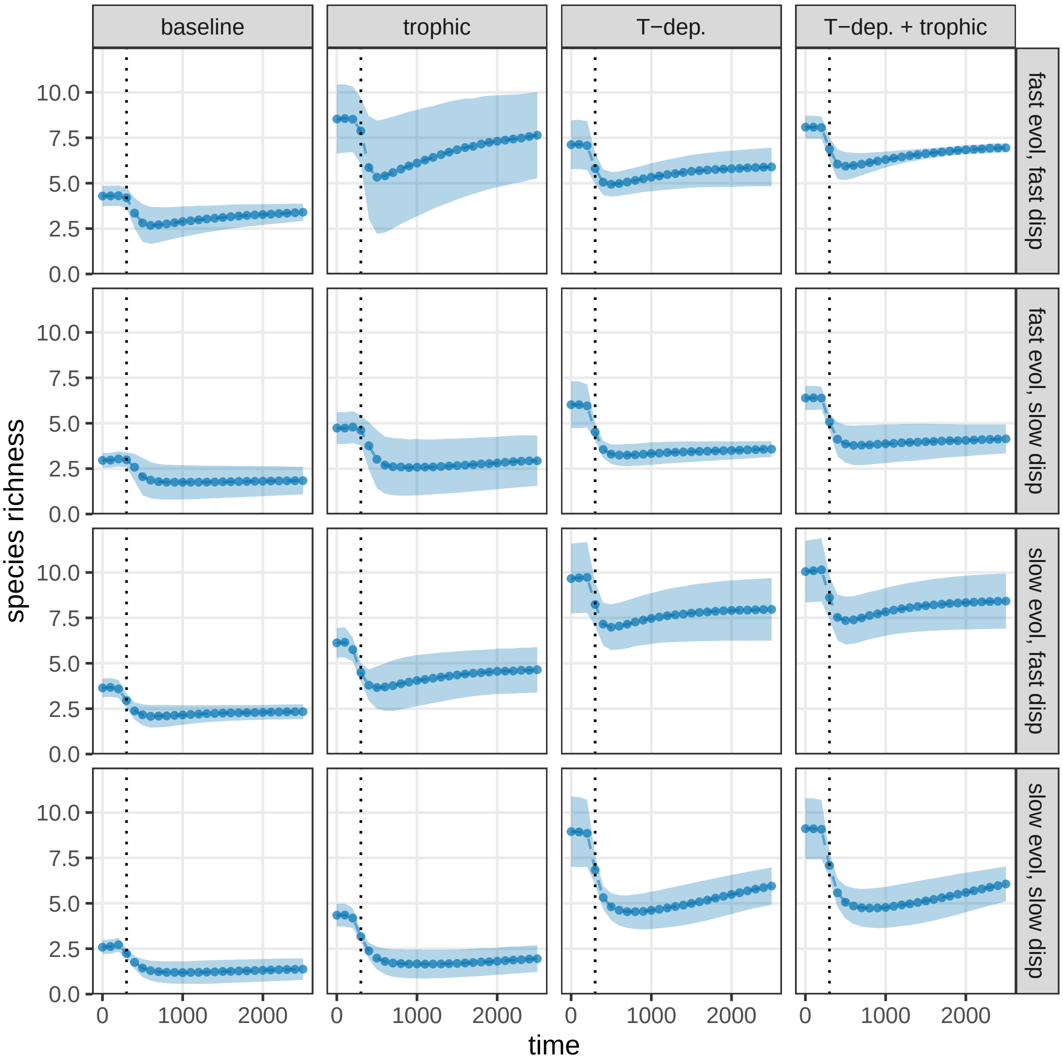

Supplementary Figure 47: As Figure 4 in the main text, but with 100 patches across the landscape instead of 50, and 20 replicates per model setup and parameterization instead of 100.

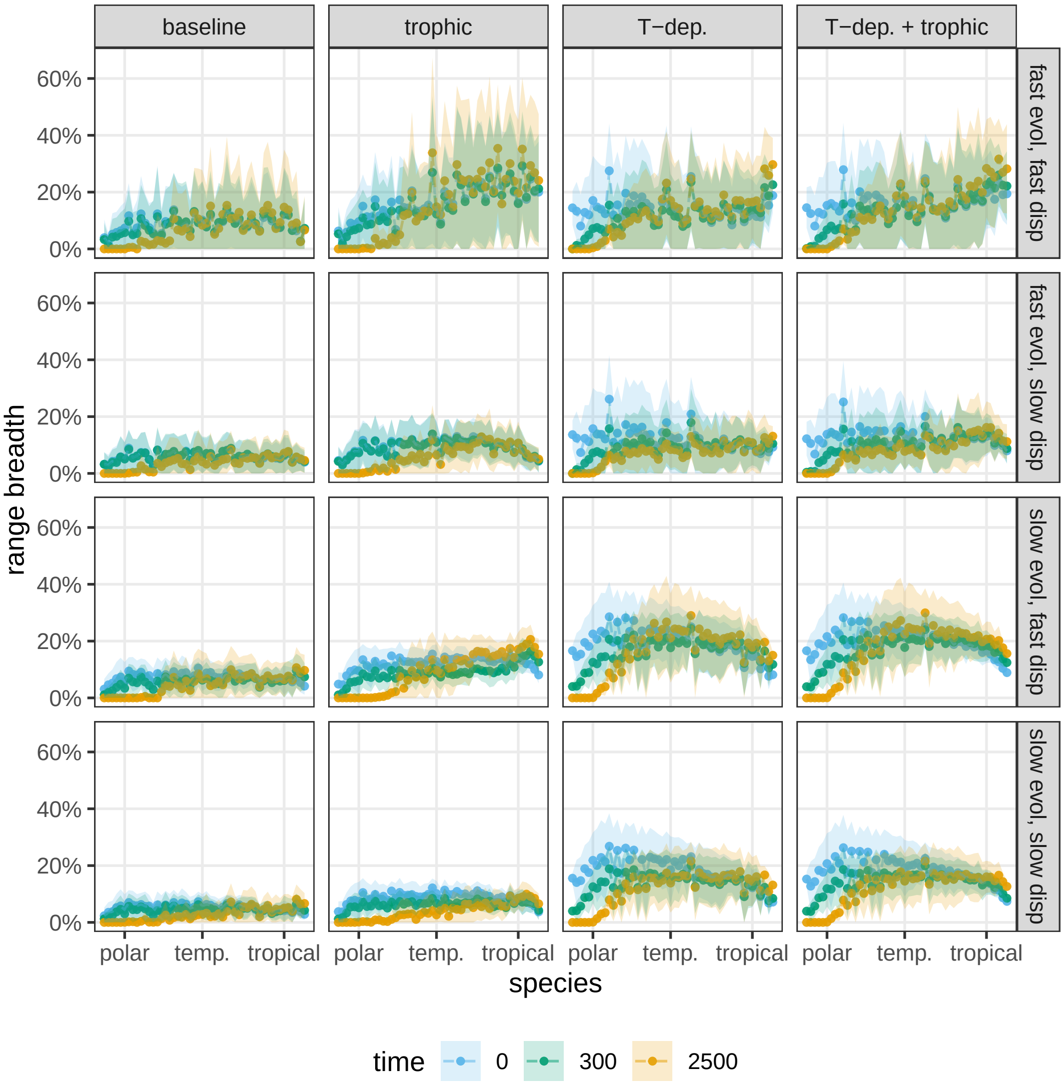

Supplementary Figure 48: As Figure 5 in the main text, but with 100 patches across the landscape instead of 50, and 20 replicates per model setup and parameterization instead of 100.

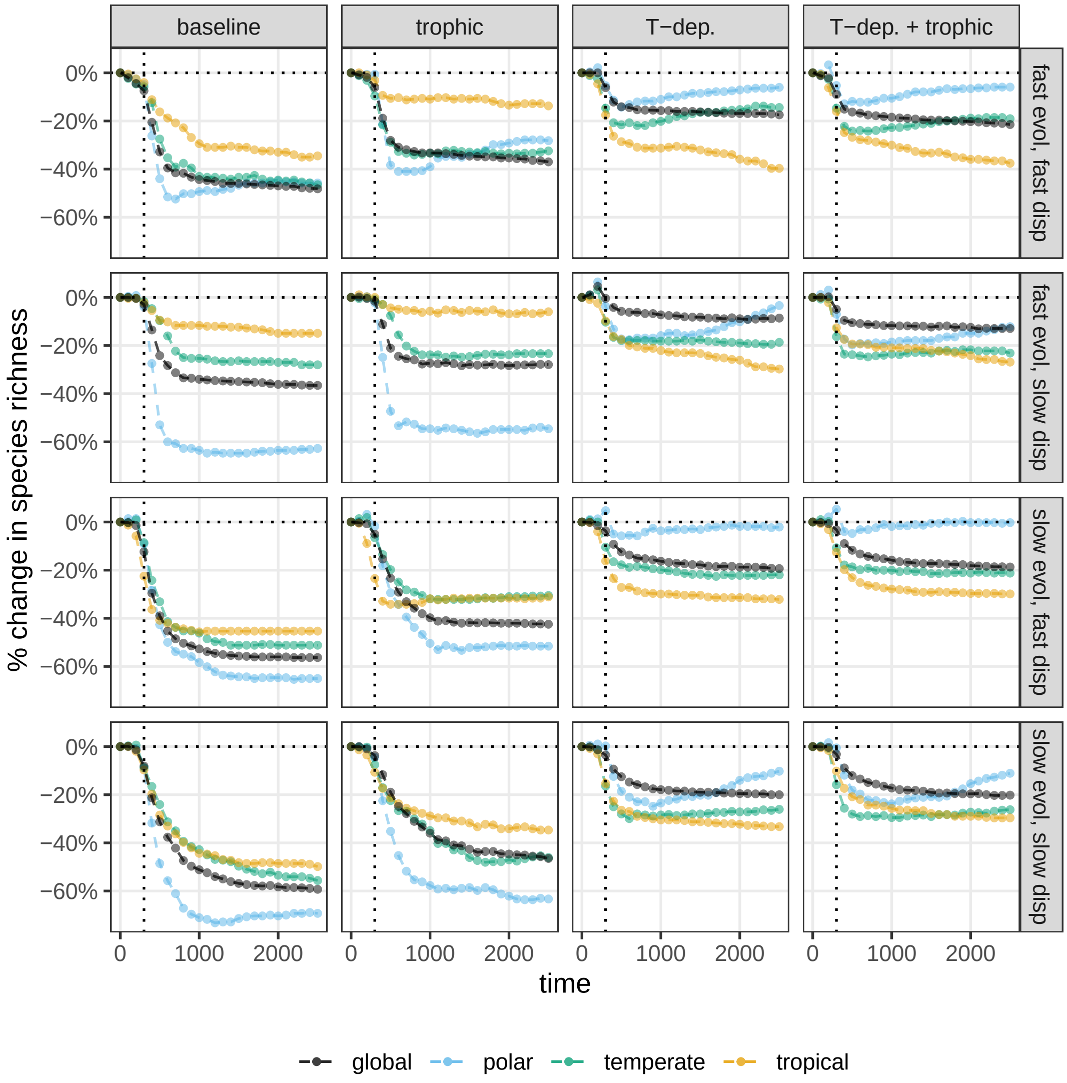

Supplementary Figure 49: As Figure 6 in the main text, but with 100 patches across the landscape instead of 50, and 20 replicates per model setup and parameterization instead of 100.

6 Parameter values

parameter	value	units	comment
S	50 / 50		no. of species (resources / consumers)
L	50		no. of patches along latitudinal gradient
X	10^7	m	distance from pole to equator
ϱ_i	[0.9, 1.1] / [0.09, 0.11]	°C yr^{-1}	tradeoff parameter (resources / consumers)
b_w	4.0	°C	tolerance width's trait-dependence intercept
a_w	0.1		tolerance width's trait-dependence slope
κ_i	0.1	yr^{-1}	intrinsic mortality
η	1	°C	competition width
ϵ_i	0.3		consumer conversion efficiency
q_i	[1, 10]	$\mathrm{g}^{-1}\mathrm{yr}^{-1}$	attack rate
H_i	[0.5, 1]	yr	handling time
W_{ij}	1 if i eats j; else 0		adjacency matrix of feeding network
ω_{ij}	[# of i's resources]$^{-1}$		relative consumption rate of i on j
$\overline{V}$	10^{-1} or 10^{-3}	°C^2	mean genetic variance
$\overline{d}$	100 or 0.01	m yr^{-1}	mean dispersal rate
a_{ii}	[0.1, 0.3]	$\mathrm{g}^{-1}\mathrm{yr}^{-1}$	intraspecific comp. coeffs. (resources only)
$a_{ij,\, j \neq i}$	[0.075, 0.225]	$\mathrm{g}^{-1}\mathrm{yr}^{-1}$	interspecific comp. coeffs. (i, j resources)
N_c	10^{-5}		threshold density for heritability reduction
$T_{\min}$	-10	°C	initial mean temperature at pole
$T_{\max}$	25	°C	initial mean temperature at equator
$C_{\min}$	1.26	°C	projected temperature increase at pole
$C_{\max}$	9.66	°C	projected temperature increase at equator
t_0	-4000	yr	establishment period before climate change
t_E	300	yr	end of climate change (starts at $t = 0$)

Supplementary Table 1: Parameter values. Unit abbreviations: g (grams), m (meters), °C (Celsius degrees), yr (years). When intervals are shown, values are uniformly drawn for each species.

parameter	value	units	comment
$V_{G,i}$	$[0.5\overline{V},\, 1.5\overline{V}]$	°C^2	genetic variance
$V_{E,i}$	$\overline{V}$	°C^2	environmental variance
σ_i^2	$V_{G,i} + V_{E,i}$	°C^2	total phenotypic variance
h_i^2	$V_{G,i}/\sigma_i^2$		heritability
d_i	$[0.1\overline{d},\, 10\overline{d}]$	m yr^{-1}	dispersal rate
m_i^{kl}	$\begin{cases} d_i & \text{if } k = l \pm 1 \\ 0 & \text{otherwise} \end{cases}$	m yr^{-1}	dispersal matrix (for k, l in $1, 2, \ldots, L$)

Supplementary Table 2: Derived parameters, using the values in Supplementary Table 1. Notation and unit abbreviations are as in Supplementary Table 1.

References

[1] R. Lande. Natural selection and random genetic drift in phenotypic evolution. *Evolution*, 30:314–334, 1976.

[2] M. Slatkin. Ecological character displacement. *Ecology*, 6:163–177, 1980.

[3] M. L. Taper and T. J. Case. Quantitative genetic models for the coevolution of character displacement. *Ecology*, 66:355–371, 1985.

[4] M. L. Taper and T. J. Case. Models of character displacement and the theoretical robustness of taxon cycles. *Evolution*, 46:317–333, 1992.

[5] G. Barabás and R. D'Andrea. The effect of intraspecific variation and heritability on community pattern and robustness. *Ecology Letters*, 19:977–986, 2016.

[6] R. Bürger. Some mathematical models in evolutionary genetics. In FACC Chalub and J. F. Rodrigues, editors, *The Mathematics of Darwins Legacy*, pages 67–89. Birkhäuser, Basel, Germany, 2011.

[7] N. H. Barton, A. M. Etheridge, and A. Véber. The infinitesimal model: Definition, derivation, and implications. *Theoretical Population Biology*, 118:50–73, 2017.

[8] M. Turelli. Commentary: Fisher's infinitesimal model: A story for the ages. *Theoretical Population Biology*, 118:46–49, 2017.

[9] D. S. Falconer. *Introduction to Quantitative Genetics*. Longman, London, UK, 1981.

[10] P. Amarasekare and C. Johnson. Evolution of thermal reaction norms in seasonally varying environments. *American Naturalist*, 189:E31–E45, 2017.

[11] A. Addo-Bediako, S. L. Chown, and K. J. Gaston. Thermal tolerance, climatic variability and latitude. *Proceedings of the Royal Society B*, 267:739–745, 2000.

[12] J. M. Sunday, A. E. Bates, and N. K. Dulvy. Global analysis of thermal tolerance and latitude in ectotherms. *Proceedings of the Royal Society B*, 278:1823–1830, 2011.

[13] C. A. Deutsch, J. J. Tewksbury, B. B. Huey, K. S. Sheldon, C. K. Ghalambor, D. C. Haak, and P. R. Martin. Impacts of climate warming on terrestrial ectotherms across latitude. *Proceedings of the National Academy of Sciences USA*, 105:6668–6672, 2008.

[14] Mridul K. Thomas, Colin T. Kremer, Christopher A. Klausmeier, and Elena Litchman. A global pattern of thermal adaptation in marine phytoplankton. *Science*, 338(6110): 1085–1088, 2012.

[15] IPCC. *Climate Change 2013: The Physical Science Basis. Contribution of Working Group I to the Fifth Assessment Report of the Intergovernmental Panel on Climate Change*, chapter 12. Cambridge University Press, 2013.

[16] J. S. Clark, D. M. Bell, M. H. Hersch, M. C. Kwin, E. Moran, A. S. Salk, et al. Individual-scale variation, species-scale differences: inference needed to understand diversity. *Ecology Letters*, 14:1273–1287, 2011.

[17] R. H. Whittaker. Evolution and measurement of species diversity. *TAXON*, 21(2-3):213–251, 1972.

[18] Matthew M. Osmond, Sarah P. Otto, and Christopher A. Klausmeier. When predators help prey adapt and persist in a changing environment. *The American Naturalist*, 190(1):83–98, 2017.

[19] Michael H. Cortez and Masato Yamamichi. How (co)evolution alters predator responses to increased mortality: extinction thresholds and hydra effects. *Ecology*, 100(10):e02789, 2019.
